# PCR‐based species identification tools for wireworms (Coleoptera: Elateridae) of economic importance in Canada

**DOI:** 10.1002/ps.71036

**Published:** 2026-06-25

**Authors:** Kathleen Furtado, Willem G van Herk, Simran Kaur Cheema, Carol E Ritland, Richard C Hamelin, Michelle T Franklin

**Affiliations:** ^1^ Agassiz Research and Development Centre, Agriculture and Agri‐Food Canada Agassiz Canada; ^2^ Department of Forest and Conservation Sciences, Faculty of Forestry and Environmental Stewardship University of British Columbia Vancouver Canada

**Keywords:** click beetle, Elateridae, integrated pest management, molecular identification, primer design, species identification tools

## Abstract

**BACKGROUND:**

Coexistence of pest and non‐pest wireworms (Coleoptera: Elateridae) in agricultural fields makes species‐level identification critical to determine when pest management measures are required. However, morphological identification of wireworms (larval stage of click beetles) is challenging, as larvae are difficult to distinguish based on morphological features and misidentifications are common. Here, we developed species‐specific primers for 15 click beetle species to be used in PCR‐based species‐level identification for agricultural fields across Canada.

**RESULTS:**

Partial sequences of the gene regions cytochrome c oxidase I (COXI), 16S, 12S, 28S, 18S, internal transcribed spacer 2 (ITS2), cytochrome‐b (CYTB), elongation factor 1 (EF1), ATP6/8, NADH dehydrogenase 1 (ND1), NADH dehydrogenase 2 (ND2), NADH dehydrogenase 3 (ND3), NADH dehydrogenase 4 (ND4), NADH dehydrogenase 5 (ND5) and NADH dehydrogenase 6 (ND6) were generated for elaterid species of interest. Of these gene regions, primers were designed on the mitochondrial gene regions COXI, CYTB and ND1 that had sufficient variation to discriminate among species and tested for species specificity using additional pest and non‐pest species from the families Elateridae, Carabidae, Scarabidae and Silphidae. Specificity testing confirmed that all primer sets were species‐specific.

**CONCLUSION:**

The novel primers designed in this study allow for PCR‐based species identification of 15 economically important click beetle pest species in Canada. Further testing is needed to validate the assay for use outside of Canada. Accurate species‐level identification will benefit pest management professionals by informing management decisions and reducing the use of insurance insecticide applications due to difficulties with identifications of wireworm pest species. © 2026 His Majesty the King in Right of Canada and The Author(s). *Pest Management Science* published by John Wiley & Sons Ltd on behalf of Society of Chemical Industry. Reproduced with the permission of the Minister of Agriculture and Agri‐Food.

## Graphical abstract

Species‐specific PCR primers and PCR‐based assays were designed for the identification of 15 economically important and difficult to distinguish wireworm pests in Canada. PCR specificity testing and performance assessments confirm the accuracy of the assays.

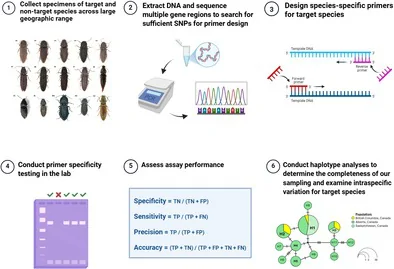

## 1 INTRODUCTION

Accurate identification of pest species is fundamental to effective integrated pest management (IPM) decisions.^1^ Rapid species‐level identifications are important to enable timely decision‐making; however, this becomes challenging for species that are morphologically difficult to distinguish.^2^ Accurate morphological identification requires time and specialized expertise, and can be difficult even for experts when species lack clear diagnostic features.^2^, ^3^ Molecular tools that enable species‐level differentiation are valuable in such cases and have been developed for cryptic and hard to‐distinguish taxa, including agricultural pests such as thrips [e.g., *Thrips tabaci*, (Lindeman), *Thrips hawaiiensis* (Morgan) and *Frankliniella intonsa* (Trybom)] in Italy and Taiwan,^2^, ^4^ potential cryptic midge species [*Dasineura oxycoccana* (Johnson)] in Canada,^5^ and the Asian and European spongy moths [*Lymantria dispar japonica* (Motschulsky), *Lymantria dispar asiatica* (Vnukovskij) and *Lymantria dispar dispar* (L.)] in Canada.^6^ To develop these tools, DNA sequences are used to develop unique oligonucleotide primer sets for each species that can be used to amplify DNA using polymerase‐chain reaction (PCR).^7^, ^8^ The mitochondrial cytochrome c oxidase 1 (COXI) gene is widely used in such studies as it is the standard barcode for species‐level identification of insects due to its reliability and robustness with over 4.2 million barcodes existing in the Barcode of Life Data System database (BOLD).^9^, ^10^

Wireworms (Coleoptera: Elateridae) are pests of potatoes, cereals and other staple field crops and can be difficult to distinguish based on morphology.^11^ Pest wireworms are generalists and feed on roots, seeds, stems and harvestable plant parts, causing damage and poor crop quality.^12^ There are few, if any, standardized scouting methods for wireworms and click beetles in North America, and no established economic thresholds for their management.^13^ Historically, pest wireworms were identified using morphological keys (e.g., Glen *et al*.^14^). Limitations of this approach include the lack of clear diagnostic features to identify pest from non‐pest larvae or from other insects commonly mistaken for pest wireworms, particularly for an inexperienced personnel.^15^, ^16^, ^17^ Additionally, the larval stage of some pest species [e.g., *Agriotes pubescens* (Melsheimer)] and most non‐pest species occurring on farmland in Canada have not yet been described.^18^, ^19^ Previous studies have developed molecular tools for accurate species‐level identification of wireworms in China, Japan and the USA.^7^, ^15^, ^20^ DNA primers for PCR assays have also been developed for pest wireworm complexes including *Agriotes* spp. in Central Europe and *Limonius* spp. in Washington, USA.^21^, ^22^

**Figure 1:**
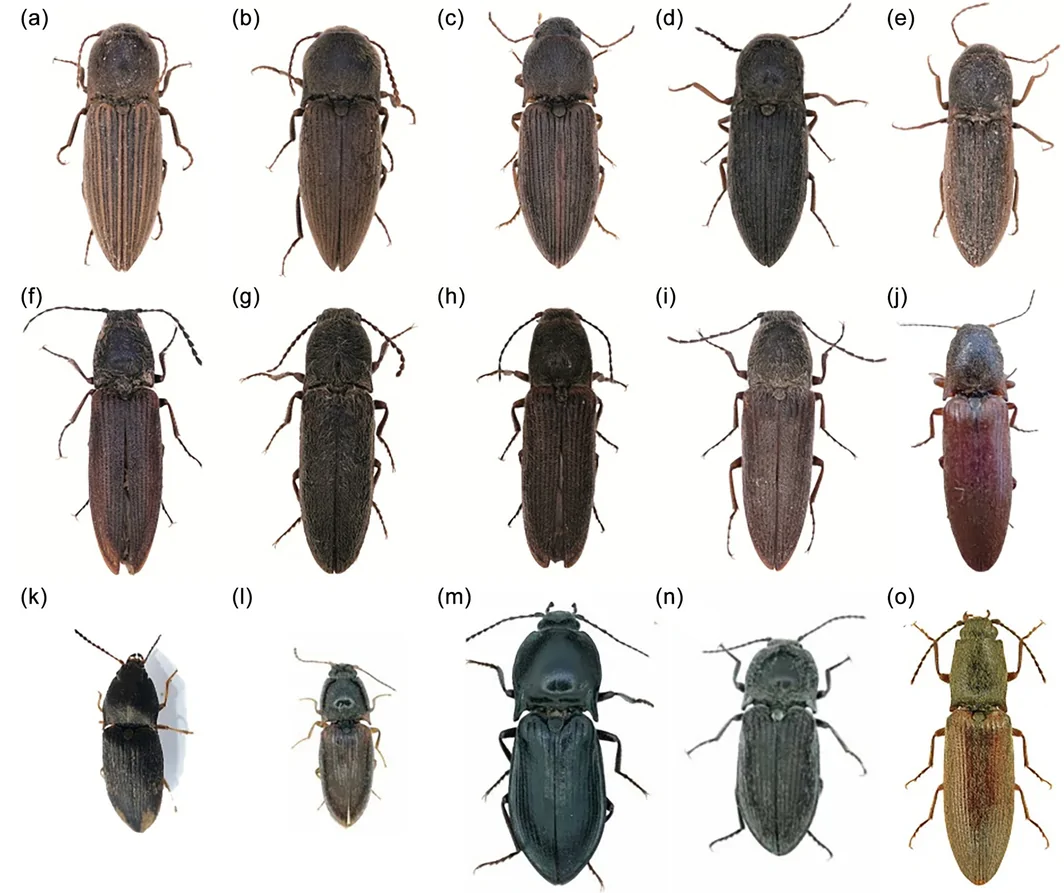
Adult click beetles of (a) *Agriotes lineatus*, (b) *A. obscurus*, (c) *A. mancus*, (d) *A. pubescens*, (e) *A. sputator*, (f) *Limonius canus*, (g) *L. californicus*, (h) *L. infuscatus*, (i) *L. agonus*, (j) *A. ferrugineipennis*, (k) *Hypnoidus abbreviatus*, (l) *H. bicolor*, (m) *Selatosomus aeripennis destructor*, (n) *Hadromorphus glaucus*, and (o) *Athous haemorrhoidalis*. Photo credits: Julien Saguez, Roxanne Sarah Bernard and Ellyne Geurts, via iNaturalist, licensed under CC0.^16^, ^59^, ^60^, ^61^

Approximately 400 click beetle species are recorded in Canada, of which about 20 are common, local or occasional pests (Table 1).^11^, ^23^ The distribution of pest and non‐pest species differs between agricultural regions.^11^ Of the latter, some species such as *Agriotes limosus* (LeConte), *Agriotes sparsus* (LeConte), *Agriotes criddlei* (Van Dyke) and various *Dalopius* spp. were once considered pests,^14^ but are likely no longer of economic importance as they were largely displaced by invasive Eurasian species that arrived over 100 years ago in Canada [*Agriotes lineatus* (L.), *Agriotes obscurus* (L.) and *Agriotes sputator* (L.)] and/or reduced in numbers due to changes in agricultural practices.^18^, ^24^, ^25^ Other species found on farmland, such as *Ampedus* spp., are likely saprophytic and do not feed on food crops.^18^ Additionally, the larvae of *Agriotes stabilis* (LeConte), *Hemicrepidius memnonius* (Herbst) and *Aeolus mellillus* (Say) are likely all occasional pests of crop plants that also reduce populations of other crop pests by predation.^26^ Unfortunately, field surveys and management of pest elaterids are constrained by the difficulty of accurately identifying and differentiating pests from non‐pest species (Fig. 1).^19^

**Table 1:** Click beetle species in the major agricultural areas of Canada, based on both historical surveys such as Glen *et al.* ^14^ and Wilkinson,^23^ and recent surveys such as Saguez *et al.*,^49^ van Herk *et al.* ^54^ Smith *et al.* ^29^

Species	West of Rocky Mountains (BC)	East of Rocky Mountains (BC and AB)	Canadian grasslands (AB, SK, MB)	Ontario	Quebec	Maritime provinces
*Athous haemorrhoidalis* (Fabricius, 1801)* ^,^ ^†^	**×**			**×**		
*Aeolus mellilus* (Say, 1834)^‡^	**×**		**×**	**×**	**×**	**×**
*Agriotes ferrugineipennis* (LeConte, 1861)^‡^	**×**					
*Agriotes lineatus* (Linnaeus, 1767)*	**×**					**×**
*Agriotes mancus* (Say, 1823)				**×**	**×**	**×**
*Agriotes obscurus* (Linnaeus, 1758)*	**×**					**×**
*Agriotes pubescens* (Melsheimer, 1845)				**×**	**×**	
*Agriotes sputator* (Linnaeus, 1758)*					**×**	**×**
*Corymbitodes lobatus* (Eschscholtz, 1822)^‡^	**×**					
*Hadromorphus glaucus* (Germar, 1843)^‡^			**×**			
*Hemicrepidius memnonius* (Herbst, 1806)^‡^			**×**	**×**	**×**	**×**
*Hypnoidus abbreviatus* (Say, 1823)				**×**	**×**	**×**
*Hypnoidus bicolor* (Eschscholtz, 1829)		**×**	**×**	**×**		
*Limonius agonus* (Say, 1839)				**×**	**×**	
*Limonius californicus* (Mannerheim, 1843)	**×**		**×**			
*Limonius canus* (LeConte, 1853)	**×**					
*Limonius infuscatus* (Motschulsky, 1859)	**×**					
*Melanotus communis* (Gyllenhal, 1817)^‡^				**×**	**×**	
*Melanotus depressus* (Melsheimer, 1844)^‡^				**×**	**×**	
*Melanotus similis* (Kirby, 1837)				**×**	**×**	
*Selatosomus aeripennis destructor* (Brown, 1935)		**×**	**×**			

* Invasive species.

^†^ Not a pest.

^‡^ Minor, or local pest.

The paucity of tools to quickly and reliably identify pest species in Canada has resulted in widespread and often unnecessary use of insecticides for their control. For example, on the Canadian Prairies, wireworm infestations are the most common reason growers use insecticide‐treated seed in cereals,^16^ and in Quebec a study suggests that neonicotinoid seed treatments in corn and soybean fields are useful in under 5% of applications due to the low pest populations or even the failure of detecting pest species.^27^ Insecticide overuse, besides being economically disadvantageous to producers, is concerning as it (e.g., neonicotinoids) has been linked to toxic effects on non‐target species such as pollinators, natural insect enemies, aquatic insects and potentially humans.^28^ Hence an improved understanding of the wireworm species complex in field crops, combined with accurate tools to distinguish pest from non‐pest species, will aid in the implementation of effective control measures for species present.^1^ This could ultimately reduce unnecessary pesticide use and its harmful environmental impacts.^29^

Efforts to develop species‐specific primers for key Canadian elaterid pest species were initiated after diagnostic primers designed by Staudacher *et al*.^21^ and Serrano *et al*.^22^ failed to separate the pest *Agriotes* and *Limonius* species, respectively, in western Canada. This was attributed to differences in the geographic location of the specimens used to develop these primers and the location of the specimens we tested them on.^30^ Additionally, the closely related and native non‐target *Agriotes* species in Canada were not considered in the design of the initial European species primers.^21^ Problems with barcode identification for species that have been under sampled across their geographic range have been highlighted previously for *Quercus* moths in Europe^31^ and deer ticks [*Ixodes scapulraris* (L.)] in North America.^32^ Diagnostic primers need to be developed so they are general enough to identify a species across a geographic range, regardless of intraspecific genetic variation, while remaining species‐specific. Considering these species frequently co‐occur with other pest species, the objective of this study was to design species‐specific primers based on mitochondrial sequence analysis and conduct PCR assays to reliably identify and differentiate between 15 important elaterid pest species across their geographic range in Canada.

## 2 MATERIALS AND METHODS

### 2.1 Specimens

**Figure 2:**
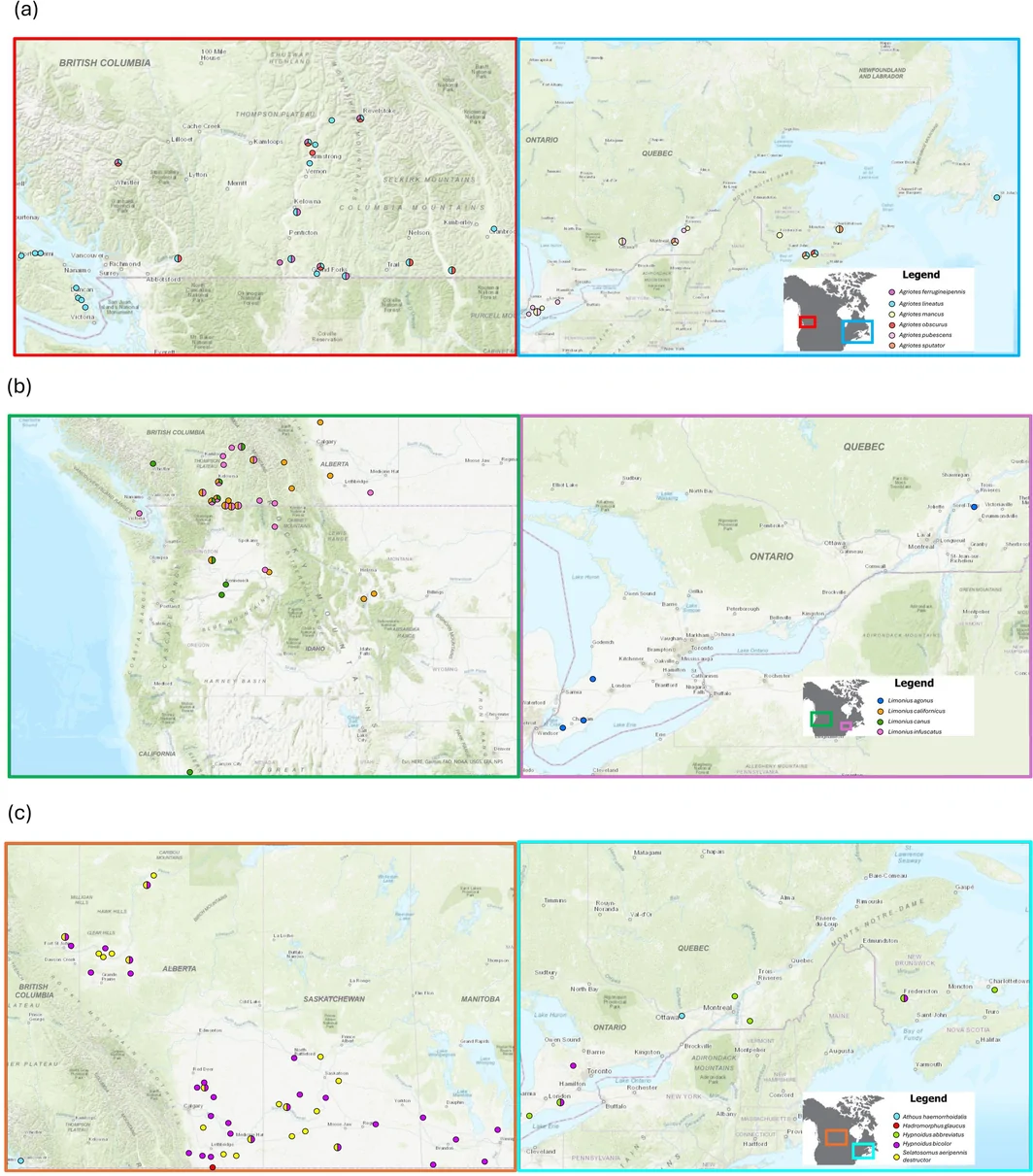
Maps of collection sites for the target species (a) *Agriotes lineatus*, *A. obscurus*, *A. sputator*, *A. mancus*, *A. pubescens* and *A. ferrugineipennis*, (b) *Limonius canus*, *L. infuscatus*, *L. californicus* and *L. agonus*, and (c) *Hypnoidus bicolor*, *H. abbreviatus*, *Selatosomus aeripennis destructor*, *Athous haemorrhoidalis* and *Hadromorphus glaucus* that were used for primer specificity testing and primer development.

Fifteen target and 35 non‐target species were collected from 119 sites across Canada, and nine sites in the USA (Supporting Information, Table S1 and Fig. 2). Target and non‐target specimens (adult and larvae) were sourced from expertly identified pinned museum specimens or preserved specimens, collected from 1956 to 2025, and stored at room temperature in 95% ethanol at the Agassiz Research and Development Centre (Agassiz, BC, Canada). Additional adult beetles were collected live from pheromone‐baited Vernon pitfall traps^33^ (VPTs; available from Intko Supply Ltd., Chilliwack, BC, Canada) or by sweep netting in the summer of 2023 and 2024. Pitfall traps were baited with the synthetic sex pheromone of *Agriotes mancus* (Say),^34^ *A. lineatus* (L.),^35^ *A. obscurus* (L.),^36^ *A. sputator* (L.),^35^ *A. ferrugineipennis* (LeConte)^37^ or limoniic acid, which attracts all four *Limonius* species.^38^ Field‐collected specimens were identified using taxonomic keys^14^, ^26^, ^39^ by K. Furtado, W. van Herk and H. Douglas. Specimens were stored at room temperature in 95% ethanol or flash frozen with liquid nitrogen and stored at −80 °C, with voucher specimens kept at the Agassiz Research and Development Centre.

### 2.2 DNA extraction

Total DNA was extracted using the DNeasy Blood and Tissue Kit (Qiagen, Hilden, Germany) from the dissected thorax or leg of the adult beetles, or the head and first two segments of larvae to avoid contamination from the gut contents and potential PCR inhibitors. The following modifications were made to the protocol: the leg or thorax was ground for 1 min with a stainless‐steel bead in the lysing Proteinase K solution with a Tissue Lyser LT (Qiagen) or Retsch MM300 Tissue Lyser (Qiagen) and incubated for 5 h, 4 μg Poly(A) RNA (Sigma‐Aldrich, Mannheim, Germany) was added to each sample to increase recovery of DNA yield, and extracted DNA was eluted in 100 μL (2 × 50 μL elution) Buffer AE. Extractions were performed manually or automated on the QIAcube Connect (Qiagen protocol ‘Tissue and Rodent Tails’ with modified elution volume). The DNA concentration of each sample was quantified using a Qubit 4 fluorometer (Thermo Fisher Scientific, Eugene, OR, USA) and quality measured through a Nanodrop 2000 Spectrophotometer (Thermo Fisher Scientific).

### 2.3 PCR and sequencing

Partial sequences of the gene regions, cytochrome c oxidase I (COXI), 16S, 12S, 28S, 18S, internal transcribed spacer 2 (ITS2), cytochrome‐b (CYTB), elongation factor 1 (EF1), ATP6/8, NADH dehydrogenase 1 (ND1), NADH dehydrogenase 2 (ND2), NADH dehydrogenase 3 (ND3), NADH dehydrogenase 4 (ND4), NADH dehydrogenase 5 (ND5) and NADH dehydrogenase 6 (ND6) were amplified for select specimens (see Supporting Information, Table S1) using the primers listed in Table 2 to generate sequence data to identify gene regions with species informative single nucleotide polymorphisms (SNPs). Primers were designed for amplification of the following gene regions: ATP6/8, ND1, ND2, ND3, ND4, ND5, ND6 from the mitochondrial genomes of *Limonius californicus* (Mannerheim)^40^ and *A. lineatus* ^41^ (Geneious Prime Version 2024.0.7). PCR reactions for all primer combinations used 2 to 42.4 ng of DNA, 5× PCR buffer (Thermo Fisher Scientific), 0.4 units of Phusion High‐Fidelity DNA polymerase (Thermo Fisher Scientific), 0.01 nmols of each primer, 0.004 μmol of dNTPs (Thermo Fisher Scientific), 4 μmol of Betaine (Thermo Fisher Scientific) to relax DNA and 1.8 μL of UltraPure DNase/RNase‐Free Distilled Water (Invitrogen, Waltham, MA, USA) in a total reaction volume of 20 μL. For all PCR reactions, a negative control of Ultrapurewater (Invitrogen) was included. The PCR conditions were as follows: 98 °C for 30 s, cycled 38 cycle times at 98 °C for 10 s, 47–52.7 °C (see Table 2 for details) for 30 s, 72 °C for 30 s, with a final extension of 72 °C for 10 min. PCR products were visualized on 1% agarose gels.

**Table 2:** The gene regions explored for species‐specific primer design alongside their respective forward and reverse PCR primers, sequences (5′ to 3′) and melting temperatures (°C) used for amplification

Gene region	Forward primer name	Forward primer sequence (5′ to 3′)	Reverse primer name	Reverse primer sequence (5′ to 3′)	Melting temperature (°C)
COXI	LCO1490^57^	GGTCAACAAATCATAAAGATATTGG	HCO2198^57^	TAAACTTCAGGGTGACCAAAAAATCA	55
16S/12S	16c^58^	CGGTGTTATCCCTAAGGT	12SB^58^	AAACTAGGATTAGATACCC	53.5
		CCCTGATACCCAGGTAC		AAACTAGGATTAGATACCC	50
			12SB^58^		
	16y^58^				
28S	28Sff^56^	TTACACACTCCTTAGCGGAT	28Sdd^56^	GGGACCCGTCTTGAAACAC	61
18S	18a2.0^56^	ATGGTTGCAAAGCTGAAAC	183'l^56^	CACCTACGGAAACCTTGTTACGAC	62
ITS2	ITS3^55^	GCATCGATGAAGAACGCAGC	ITS4^55^	TCCTCCGCTTATTGATATGC	60.4
CYTB	REVCB2H^6^	TGAGGACAAATATCATTTTGAGGW	REVCBJ^6^	ACTGGTCGAGCTCCAATTCATGT	60
EF1	EF1_Starsky_For^6^	CACATYAACATTGTCGTSATYGG	EF1_Luke_Rev^6^	CATRTTGTCKCCGTGCCAKCC	59
ATP6/8	ATP8/ATP6‐KF1*	ATTAACTGAAAATGATAA	ATP8/ATP6‐KF5*	CCWGCAATTATATTWGC	47
ND1	tr/NAD1‐KF*	CATWTAAAGATTCTAAATCAAT	ND1‐KF‐F2*	ACTCCNTTTGATTTTGC	51.8
ND2	ND2‐KF4*	TAGGAATATGRCTAGG	ND2‐KF1*	TGRATTGTTAATCATTTWGG	47
ND3	CO3/ND3F‐KF2*	TCTAYTGATGAGGTAGWT	tR‐Asn/NAD3R‐KF*	TATCATTAACAGTGATAWA	49.3
ND4	ND4L‐KF‐R1*	CRTCACARACTCTTATAGT	ND4‐KF4*	TAAGTCTTTAATTGCTTATTC	52.7
ND5	NAD5‐KF12*	ACTAAACAATAAGAAACRA	NAD5‐KF1*	TTGGTTATGAGTTTTCWAA	49.4
ND6	tR/NAD6F‐KF*	CTTTTAAAACTTCAGAGA	CYTb/NAD6R‐KF*	GTWATTTTWACWACTGC	47.6

Primers used were either existing primers from Stewart *et al*.,^6^ Yao *et al*.,^55^ Kundrata *et al*.,^56^ Folmer *et al*.^57^ and Gunter *et al*.^58^ or designed in this study from *Limonius californicus* ^40^ and *Agriotes lineatus* ^41^ reference genomes.

* Primers designed in this study.

PCR products were purified using ExoSAP‐IT Express (Applied Biosystems, Foster City, CA, USA) as per manufacturer's recommendations. Sanger sequencing reactions were performed with BigDye Terminator v3.1 Cycle Sequencing Kit (Applied Biosystems) using the forward and reverse primers. A modification of 1 μL of BigDye Terminator for a 10‐μL reaction mix was made to the manufacturer's recommendations, as in‐lab testing determined that this was sufficient for generating Sanger fragments. Reaction products were purified using BigDye XTerminator Purification Kit (Applied Biosystems) and subsequently sequenced on SeqStudio Genetic Analyzer (Applied Biosystems) in both forward and reverse directions.

Consensus sequences were generated and aligned in Geneious Prime (version 2024.0.7) and analysed for the number of single nucleotide polymorphisms (SNPs) in each gene region. BLASTn search was performed for COXI sequences to determine if the morphological species identification matched with the maximum BLAST scores of 100% coverage and 98–100% identity. Due to the presence of haplotypes in some of our sequences that were not present in GenBank or BOLD, 98% identity was used for the matching sequence.^42^ Sequences were deposited in BOLD (CBAGA001‐24 to CBAGA325‐25). Intraspecific variation was determined for the gene regions used in primer design (COXI, CYTB and ND1) by analysing pairwise identity in Geneious Prime.

### 2.4 Development of species‐specific primers

A total of 544 sequences from 308 target and non‐target beetles were analysed for the development of species‐specific primers. Species‐specific primers were designed from the COXI region for all species, with the exception of *A. lineatus* and *A. obscurus* where primers were designed from the CYTB and ND1 gene regions due to a low abundance of species informative SNPs present at the COXI region. Primer design tools (Geneious Prime 2024.0.7) were used to design primers using the criteria of Saccaggi *et al*.^43^ The melting temperatures (Tm) of candidate primer pairs were calculated to ensure that the melting temperatures of the forward and reverse primers were between 5 °C of each other and at least 5 °C below the annealing temperature.

### 2.5 Primer specificity testing

Candidate primer combinations were tested for amplification of the target pest species using DNA extracted from three individuals per collection site (Supporting Information, Table S1). An additional 13 to 28 non‐target pest and non‐pest species were tested for primer specificity from DNA extracted from two to five individuals per collection site (Supporting Information, Table S1). Non‐target species selected from the beetle families Elateridae, Carabidae, Scarabidae and Silphidae either coexist alongside the target species of interest or are closely related and belong to the same genus. Annealing temperatures ranging from 55 °C to 66 °C were tested for each primer combination to obtain an optimal melting temperature (Tm). PCR reactions were performed with the same reaction mix described previously with the following modifications: reduced primer amount of 5 pmols and the exclusion of Betaine. PCR conditions were as follows: 98 °C for 30 s, cycled 30 cycle times at 98 °C for 10 s, 62–66 °C (see Table 3 for details) for 30 s, 72 °C for 30 s, with a final extension of 72 °C for 5 min. PCR products were visualized on 1% agarose gels.

**Table 3:** Summary of novel species‐specific PCR primers for our 15 species of interest, including the target species, gene region, primer names (F and R denote forward and reverse, respectively), primer sequences, melting temperature (°C) and expected amplicon size (bp)

Target species	Gene region	Primer name	Sequence (5′ to 3′)	Melting temperature (°C)	Amplicon size (bps)
*Agriotes lineatus*	ND1	AL‐ND1‐292F	TTTGCTTTAGTTTTATGGGTTTGTGTA	63	312
		AL‐ND1‐582R	TACCATCCCGAGAGGAATACTC		
*Agriotes obscurus*	CYTB	AO‐cytb‐123F	TACACTACACTTTTTACTACCATTTG	62	256
		AO‐cytb‐353R	AGTTACTAAAGGATTGGCAGGA		
*Agriotes sputator*	COXI	A‐spu‐F1	CCCTGGGTCATTAATCGGAAAT	63	211
		A‐spu‐R2	TAATAGAAGAGACAATGAAGGTGGAAGA		
*Agriotes mancus*	COXI	A‐man‐F2	CCCGGATCATTAATCGGAAAT	64	469
		A‐man‐R1	GTAAGGATAGGAGGAGGAGTAGG		
*Agriotes pubescens*	COXI	A‐lin/obs/pub‐F1	CCCTGGATCACTAATCGGAAAT	64	522
		A‐pub‐R2	GTGTTTAAATTTCGATCAGTAAGTA		
*Agriotes ferrugineipennis*	COXI	Ferrug‐259F	CATAAGATTCTGGTTTCTACCCCCT	65	299
		Ferrug‐537R	TGGAAGTGATAGGAGTAGAAGTAGG		
*Limonius agonus*	COXI	Agonus‐222F	TTGACTAGTACCACTTATACTAGGA	65	362
		Agonus‐544R	CTGCAAGAACTGGTAGAGAG		
*Limonius infuscatus*	COXI	Infus‐245F	CCTTCCTCCATCGCTTTCC	65	300
		Infus‐572R	CCGGCAAGAACTGGTAAGG		
*Limonius californicus*	COXI	Cali‐189F	GTTCGGAAACTGACTAGTTCCA	65	310
		Cali‐492R	ACGGTCGAAGGTAATTCCGGTA		
*Limonius canus*	COXI	Canus‐274F	CTACCCCCGTCTCTTTCCCTC	65	219
		Canus‐492R	GGTGATTCCTGTGGATCGC		
*Selatosomus aeripennis destructor*	COXI	SD‐1F	AAGATTCTGATTCCTCCCACCCT	64	292
		SD‐124R	TAAGAGTAGAAGGAGGGCTG		
*Hypnoidus bicolor*	COXI	HyB‐472F	TGGTAATTGATTAGTACCCCTCA	65	161
		HyB‐1R	AAGGAGGGTATACTGTTCAACC		
*Hypnoidus abbreviatus*	COXI	HyA‐623F	AGGAACATCCTTAAGTCTCCT	64	385
		HyA‐232R	CACTTAGCTGGAATCTCCTCT		
*Hadromorphus glaucus*	COXI	HG‐484F	CCTGGGTCTCTTATTGGAAACG	66	463
		HG‐23R	ACAATAGGAGTAGTAGGGCAGTG		
*Athous haemorrhoidalis*	COXI	Ath‐224F	ACCCTTATCAGCCAATATTGCCCAT	65	205
		Ath‐19R	AGAGATAGGAGTAGGAGGAGTGCA		

### 2.6 Performance assessment equations

The best performing primer pairs from the primer specificity testing were assessed for each target species. The following equations from Capron *et al*.^44^ were used to calculate specificity, sensitivity, precision and accuracy of each primer pair using PCR data from the primer specificity testing:

$$\text{Specificity}=\mathrm{TN}/\left(\mathrm{TN}+\mathrm{FP}\right)$$

$$\text{Sensitivity}=\mathrm{TP}/\left(\mathrm{TP}+\mathrm{FN}\right)$$

$$\text{Precision}=\mathrm{TP}/\left(\mathrm{TP}+\mathrm{FP}\right)$$

$$\text{Accuracy}=\left(\mathrm{TP}+\mathrm{TN}\right)/\left(\mathrm{TP}+\mathrm{FP}+\mathrm{TN}+\mathrm{FN}\right)$$

In the equations, TN represents a true negative, TP a true positive, FN a false negative and FP a false positive. The resulting data was converted into percentages.

### 2.7 Haplotype accumulation curves and network analysis

To determine if our sampling efforts had captured the haplotype diversity of each target species, we generated haplotype accumulation curves using HACSim.^32^ The haplotype analyses were used to determine how well our sampling effort represented the potential diversity of haplotypes for each species across their full geographic range and what sample size would be required to identify 95% of the haplotype variation. Sequences produced as part of this study for primer design and an additional 200 published sequences from NCBI (Supporting Information, Table S4) were used for this analysis. To avoid overestimations of observed haplotypes, sequences with ambiguous nucleotides were removed from the analysis.^32^ Sequences were trimmed to the same length and ran with 100 000 permutations and 95% confidence intervals.

In addition, haplotype networks were constructed using the Hapsolutely software^62^ for target species. The same sequences used for the haplotype accumulation curves were visualized as median‐joining networks. Species with only one haplotype observed were excluded from the above analyses.

## 3 RESULTS

### 3.1 Sanger sequencing

Intraspecific variation ranged from 0% to 5.1% across all geographic sites for target species based on analysis of gene regions where primers were designed (Supporting Information, Table S2). Less than 1% intraspecific variation was found for 11 out of 15 species for the COXI, 8 out of 11 species for the CYTB and 8 out of 10 species for the ND1, which supports our 98% sequence identity cutoff for species identification using COXI sequences in BLASTn. The highest intraspecific variation measured was for *Hypnoidus bicolor* (Eschscholtz) at the COXI gene with 5.1% variation. *L. californicus* exhibited the highest intraspecific variation in each gene region compared to other species (see Supporting Information, Table S2).

### 3.2 Primer specificity testing

**Figure 3:**
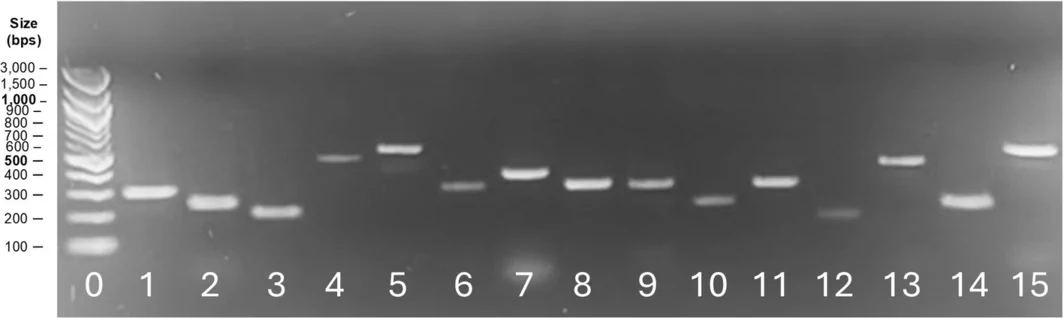
PCR products of target pest species using the successful species‐specific primer assays for the 15 target species from left to right are (0) 100 bp Plus ladder (Thermo Fisher Scientific), (1) *Agriotes lineatus* (AL‐ND1‐292F and AL‐ND1‐582R, 312 bp), (2) *Agriotes obscurus* (AO‐cytb‐123F and AO‐cytb‐353R, 256 bp), (3) *Agriotes sputator* (A‐spu‐F1 and A‐spu‐R2, 211 bp), (4) *Agriotes mancus* (A‐man‐F2 and A‐man‐R1, 469 bp), (5) *Agriotes pubescens* (A‐lin/obs/pub‐F1 and A‐pub‐R2, 522 bp), (6) *Agriotes ferrugineipennis* (Ferrug‐259F and Ferrug‐537R, 299 bp), (7) *Limonius agonus* (Agonus‐222F and Agonus‐544R, 362 bp), (8) *Limonius infuscatus* (Infus‐245F and Infus‐572R, 300 bp), (9) *Limonius californicus* (Cali‐189F and Cali‐492R, 310 bp), (10) *Limonius canus* (Canus‐274F and Canus‐492R, 219 bp), (11) *Selatosomus aeripennis destructor* (SD‐124F and SD‐1R, 292 bp), (12) *Hypnoidus bicolor* (HyB‐1F and HyB472R, 161 bp), (13) *Hypnoidus abbreviatus* (HyA‐232F and HyA‐623R, 385 bp), (14) *Athous haemorrhoidalis* (Ath‐224F and Ath‐19R, 205 bp) and (15) *Hadromorphus glaucus* (HG‐484F and HG‐23R, 463 bp).

A total of 99 primer candidates (COXI = 90 primers, CYTB = 4 primers, ND1 = 5 primers; Supporting Information, Table S3) were designed and screened for species specificity. Out of these, one pair was deemed species‐specific for each target species with amplification occurring for the target species only (Table 3, Fig. 3 and Supporting Information, Fig. S1). True positive (TP) amplification occurred across all geographic populations tested for each target species, with the exception of false negatives (FN) for 100% of *L. californicus* samples from Manhattan, Montana; George, Washington; and 33% of samples from Lenville, Idaho, and *Limonius infuscatus* (Motschulsky) samples from Manhattan, Montana; Moscow, Idaho; and 67% of Sandpoint, Idaho (Supporting Information, Fig. S4).

### 3.3 Performance assessment

Each successful primer pair had 100% specificity, sensitivity, precision and accuracy for all populations tested within Canada. Several samples tested from populations outside of Canada created false negatives (FNs) for *L. infuscatus* and *L. californicus* assays (see Section 3.2; Supporting Information, Fig. S4). Accuracy for *L. infuscatus* and *L. californicus* dropped to 98%, and sensitivity for *L. californicus* and *L. infuscatus* dropped to 90% and 88%, respectively, when considering populations outside of Canada (Table 4).

**Table 4:** Performance assessment results for specificity, sensitivity, precision and accuracy for each target species' PCR assay

Target species assay	Specificity (%)	Sensitivity (%)	Precision (%)	Accuracy (%)	*N*
*Agriotes lineatus*	100	100	100	100	293
*Agriotes obscurus*	100	100	100	100	246
*Agriotes sputator*	100	100	100	100	101
*Agriotes mancus*	100	100	100	100	168
*Agriotes pubescens*	100	100	100	100	115
*Agriotes ferrugineipennis*	100	100	100	100	128
*Limonius agonus*	100	100	100	100	117
*Limonius infuscatus*	100	88*	100	98*	315
*Limonius californicus*	100	90*	100	98*	334
*Limonius canus*	100	100	100	100	292
*Selatosomus aeripennis destructor*	100	100	100	100	117
*Hypnoidus bicolor*	100	100	100	100	135
*Hypnoidus abbreviatus*	100	100	100	100	104
*Hadromorphus glaucus*	100	100	100	100	175
*Athous haemorrhoidalis*	100	100	100	100	112

The total number of samples tested for each primer pair is reported (*N*).

* Metrics are 100% when considering only populations in Canada.

### 3.4 Haplotype accumulation curves and networks

Our sampling effort based on sequence data ranged from 2 to 112 individuals per species and was estimated to represent between 70.5% to 99.5% of all observed haplotype diversity across each target species geographic range (Supporting Information, Fig. S2 and Table 5). *Athous haemorrhoidalis* (Fabricius) was estimated to have the most sufficient sequencing sample coverage to capture haplotype variation, with the 41 samples analysed representing 99.4% of the haplotype diversity expected across this species geographic range. The sampling effort for *Limonius canus* (LeConte) based on sequence data was estimated to have the least sufficient coverage, with the 12 samples analysed representing only 70.5% of the expected haplotype diversity. For identification of at least 95% of all haplotype variation for *L. canus*, it was estimated that an additional 22 individuals would need to be sampled and sequenced to give a total of 34 samples (95% confidence interval 33.007–34.993).

**Table 5:** Haplotype analyses using DNA sequence data for each target species with multiple unique haplotypes

Species	Specimens sequenced	Unique haplotypes	Estimated proportion of haplotypes sampled (%)	Additional samples needed for 95% haplotype recovery	Total sampling sufficiency for 95% haplotype recovery	Specimens sampled for PCR testing
Agriotes lineatus*	3	1	–	–	–	92
*Agriotes obscurus*	2	5	74.9	3	5 (95% CI: 4.4546–5.5454)	20
*Agriotes sputator*	40	5	80.2	50	90 (95% CI: 88.2579–91.7421)	9
Agriotes mancus*	10	1	–	–	–	17
*Agriotes pubescens*	12	7	77.5	17	29 (95% CI: 28.1634–29.8366)	18
*Agriotes ferrugineipennis*	7	2	82.7	8	15 (95% CI: 13.8092–16.1908)	22
*Limonius agonus*	5	2	83.6	6	11 (95% CI: 10.0595–11.9405)	9
*Limonius infuscatus*	12	3	76.6	18	30 (95% CI: 28.6405–31.3595)	67
*Limonius californicus*	35	12	75	56	91 (95% CI: 89.9076–92.0924)	60
*Limonius canus*	12	6	70.5	22	34 (95% CI: 33.007–34.993)	27
*Selatosomus aeripennis destructor*	56	12	76	85	141 (95% CI: 139.603–142.397)	33
*Hypnoidus bicolor*	110	12	82.5	130	240 (95% CI: 238.09–241.91)	38
Hypnoidus abbreviatus*	21	1	–	–	–	12
Hadromorphus glaucus*	8	1	–	–	–	5
*Athous haemorrhoidalis*	41	3	99.4	0	41 (95% CI: 41–41)	9

Analyses could not be conducted for species with only one haplotype, which are noted with an asterisk (*). Columns present the species, number of specimens used in the haplotype analyses, number of unique haplotypes, estimated proportion of haplotypes sampled over the full geographic range for each species (%), additional samples needed for 95% haplotype recovery over the entire geographic range of each species, total sampling sufficiency for 95% haplotype recovery (with 95% confidence intervals) and specimens tested for primer specificity during development of PCR assays.

The greatest haplotype diversity was found for *L. californicus*, *H. bicolor* and *Selatosomus aeripennis destructor* (Brown) (Supporting Information, Fig. S3). A total of 13 haplotypes were found for *L. californicus*, of which five haplotypes were represented by more than one individual. The most common haplotype for *L. californicus* was shared by 17 individuals over two geographic sites. A total of 12 haplotypes were found for both *H. bicolor* and *S. a. destructor*. For *H. bicolor*, six haplotypes were represented by more than one individual and the most common haplotype was shared by 58 individuals over eight geographic sites. Five haplotypes were represented by more than one individual for *S. a. destructor*, with the most common haplotype represented by more than 36 individuals across three geographic sites.

## 4 DISCUSSION

We developed a series of unique PCR primers that provide a high level of specificity, sensitivity, precision and accuracy for the detection of 15 target click beetle species across their geographic ranges in Canada (see Table 3). These species represent six genera, with three being non‐native pests (*A. lineatus*, *A. obscurus* and *A. sputator*), two being minor or localized pests (*A. ferrugineipennis* and *H. glaucus*) and one non‐native species with an unknown pest status in Canada (*A. haemorrhoidalis*). Click beetles were collected and sequenced from multiple sites across Canada to be used for primer design and primer specificity testing, giving validity to the use of these primers for species identification across the geographic range of each species in Canada.^45^ Primer pairs were tested for specificity against non‐target DNA of 34 additional wireworm pest species, non‐pest wireworms and other non‐target beetle taxa. The non‐target species chosen either coexist alongside the target species of interest, or are closely related,^18^, ^23^, ^26^ indicating that the species‐specific primers developed in this study will provide robust species identification tools for these 15 target species across Canada.

Our study adds Canadian wireworm identification assays to the existing wireworm molecular identification tools that are currently available for identification of wireworm species complexes in Central Europe^21^ and in Washington State, USA.^22^ The larvae of these species, especially those belonging to the same genus, such as the four *Limonius* species, and the five *Agriotes* species in this study have similar morphologies, making them difficult to accurately identify to species.^23^, ^26^, ^38^ Rather than using multiple morphological elaterid keys to identify wireworm species, our primers allow for accurate identification of the larval stage of these pest species.^15^ As PCR methods are common and are standard across a range of laboratories, any facility equipped with a PCR thermocycler, primers and knowledge on how to use them can readily conduct these assays.

The morphological similarity of wireworms has made it difficult to determine which pest species are present and their abundance in agricultural fields.^15^, ^18^ For example, *A. pubescens* is commonly found on agricultural land in Ontario and Quebec, co‐occurring with *A. mancus*, but its pest status and life history have not been determined.^18^ Similarly, *A. ferrugineipennis* is commonly found on agricultural land in parts of southern British Columbia, but its pest status remains unstudied.^37^ Both *A. pubescens* and *A. ferrugineipennis* can be collected in large numbers with adult‐pheromone traps, potentially leading to overestimations of the risk to crop, particularly if further studies find evidence that *A. ferrugineipennis* is not, or is only a minor pest.

Using the diagnostic species‐identification tools, such as the primers described herein, will contribute to determining the presence and abundance of different pest species on farmland to inform IPM decisions, particularly when combined with absolute sampling tactics such as passive trapping of adults, soil coring or bait trapping of larvae that collect the various species present more or less equally.^11^ To use the developed tools, trapping should take place prior to planting and when wireworms are actively feeding near the soil surface. This generally occurs twice during the year: in spring when the soil surface warms to 10 °C and in early to mid‐August depending on the species and larval age.^16^ Identifying all specimens collected in these samples to species will provide a more reliable estimate of the ratio of pest to non‐pest species present, and this, combined with additional data such as field history and the crop grown, can allow growers to make more informed decisions as to the risk wireworms pose to crops prior to insecticide applications. This is important, as growers commonly apply insecticide seed treatments for wireworm management prophylactically, having few tools for determining the likelihood of crop damage.^27^, ^28^ This leads to high pesticide costs and harmful environmental impacts to non‐target species.^28^, ^29^ Wireworm or adult beetle samples collected can be given directly to diagnostic and government laboratories that adopt our tools for PCR‐based testing using the primers and protocols outlined in this study, reducing our reliance on expert taxonomic skills for identification.^18^ Expert identified voucher specimens or DNA samples can be obtained from the Agassiz Research and Development Centre.

Understanding which wireworm species are present in the field will allow producers to determine what control methods to use (insecticide, trap crop, etc), particularly as pest species vary in their response to such tactics.^18^ This will depend on how well the biology of the species is known and the availability of attractants (sex pheromones or kairomones) for these species. Work towards developing field guides has begun that will help growers determine how to sample for wireworms and click beetles, what species are present, assess the risk species pose to a crop and how they respond to different control tactics.^16^, ^49^ Sections of existingfield guides could also be updated to include sections on how to use PCR primers for species identification, including those developed in this study. Such addition would need to be region‐specific as pest species assemblages vary per province (Table 1) and would describe how to use these primers in different assays.

A common application of PCR assays is for detecting the spread of invasive insects.^46^ The developed primers in our study could be used to track the spread of *A. haemorrhoidalis*, a species recently established in western British Columbia (van Herk, unpublished data), Ontario and Massachusetts.^47^ Historically reported as a likely pest of agriculture in Europe, the establishment of *A. haemorrhoidalis* in Canada is of concern, as it is likely to continue to spread and establish.^47^ Another invasive species, *A. sputator*, has recently established in Quebec, where it coexists in crop fields with both *A. pubescens* and *A. mancus*.^24^ In Prince Edward Island, this species caused significant potato yield loss of up to 20–30% until the recent registration of new effective insecticides,^24^ and it is likely to be a pest in other areas as it continues to move west in Canada or south toward the Midwestern USA. In addition, the primers reported herein can be used to track the continued dispersal of *A. lineatus* and *A. obscurus* from coastal western Canada to further inland, at least one of which (*A. lineatus*) has now established in the East Kootenay region of southeastern British Columbia (van Herk, unpublished data), a considerable range expansion since previous surveys.^48^

Development of species‐specific primers also allows us to investigate the impact of these invasive species on native ones, especially if they have similar life histories or occupy a similar niche. Examples of where invasive wireworms have displaced native species include *A. sputator* now dominating agricultural fields in Nova Scotia instead of the historically dominant *A. mancus*,^23^, ^24^ *A. lineatus* becoming the dominant pest in Newfoundland (van Herk, unpublished data), and *A. lineatus* and *A. obscurus* having displaced once‐abundant native pest species such as *A. sparsus* and multiple *Limonius* spp. in the Fraser Valley of British Columbia.^23^

The development of these primers is the first step to a possible multiplex PCR assay that can identify multiple species in a single PCR reaction, saving time and allowing for many samples to be processed at once. Primer pairs with considerably different amplicon sizes (at least 50 bp apart) have the potential to be used in multiplex detection system and be effectively distinguished on an agarose gel.^21^ Such primer combinations would need to work under the same PCR conditions (i.e., melting temperature) and selecting them would be province specific and combined ideally by genus. For example, *A. obscurus*, *A. lineatus* and *A. ferrugineipennis* primer pairs would be combined for use in British Columbia, *A. obscurus*, *A. lineatus* and *A. sputator* for Prince Edward Island, and *A. lineatus*, *A. mancus*, *A. pubescens* and *A. sputator* for Quebec. Additionally, future applications could incorporate the use of our novel primers in field‐adapted PCR applications using approaches such as the *in Situ* Processing and Efficient Environmental Detection (iSPEED) that has been used for rapid‐field based identification of the European (*L. d. dispar*) and Asian spongy moth (*L. d. asiatica*) with a portable PCR machine.^50^ Field techniques have been proven to provide rapid, cost‐effective and reliable identifications of a target species with the use of unique primers, even on field degraded samples.^50^, ^51^

Several factors, including outstanding taxonomic questions, technical know‐how and insufficient samples, prevented the development of diagnostic primers for the remaining pest species in Canada, [e.g., multiple *Melanotus* spp., *Aeolus mellilus*, *Hemicrepidius memnonius* and *Corymbitodes lobatus* (Eschscholtz)] at this time^18^ (Table 1). This is a subject for further research. In addition, the genetic diversity and intraspecific variation of wireworm species with large distributions (e.g., *H. bicolor*) and populations with geographically isolated regions (e.g., *L. californicus, L. infuscatus, S. a. destructor*) should be explored further.^3^, ^8^, ^52^ This could allow us to understand the similarity (genetic, morphological, composition of sex pheromones) between closely related species (e.g., Nearctic *A. mancus*, *A. pubescens* and Palearctic *A. obscurus*, *A. lineatus*).^18^, ^23^, ^34^ For example, it is likely that the genetic diversity within the western *Limonius* species, as demonstrated by the haplotype analyses in this study, contributed to why primers developed for Washington and Oregon^22^ were unsuccessful for separating species collected in Canada. The number of specimens required for assay design and testing is dependent on the distribution of the populations and the magnitude of genetic diversity within populations, as there is no universal sample size that can capture most intraspecific genetic variation across species.^53^

We are confident in the use of our diagnostic primers across their tested geographic range, as they are based on specimens collected across multiple geographically separated and genetically distinct elaterid populations across Canada to account for the potential presence of cryptic species (such as for *L. californicus*, *L. infuscatus* and *H. bicolor*) and included rigorous specificity testing of non‐target species. The high intraspecific variation observed in this study for both *H. bicolor* and *L. californicus* were consistent with findings from Drahun *et al*.,^52^ Benefer *et al*.^3^ and Andrews *et al*.,^8^ who also theorize the presence of cryptic species complexes for both species. The use of haplotype accumulation curves has assisted in estimating if our sample size for each species was sufficient to capture the majority of intraspecific genetic variation for each species across its entire geographic range and highlights the need for increased availability of specimen and sequence data in public repositories such as BOLD.^32^ Based on haplotype analyses, we have reached sufficient sampling for our designed primers, based on sequencing and PCR testing efforts, to capture 95% of all intraspecific variation for each target species except *A. sputator*, *S. a. destructor* and *H. bicolor*. We cannot be confident that our primers capture the entire intraspecific variation that may be present outside of Canada as this is beyond the scope of the present study and outside of our feasible sampling area. This was observed by the decreased accuracy and sensitivity values for *L. californicus* and *L. infuscatus* primers due to false negatives for specimens collected in the USA. Therefore, caution should be taken for use of the developed assays outside of the tested geographic range for species that have not reached sufficient sampling efforts (*A. sputator*, *S. a. destructor*, *H. bicolor*) or those that provided false negatives in regions outside of Canada (*L. infuscatus*, *L. californicus*). Future research including collections across entire species ranges will help with the completion of wireworm molecular detection assays and validation of primer use outside of Canada.

## 5 CONCLUSION

In conclusion, we have developed species‐specific primers with high sensitivity, accuracy, precision and specificity on the CYTB gene for *A. obscurus*, the ND1 gene for *A. lineatus* and the COXI gene for *A. sputator*, *A. mancus*, *A. pubescens*, *A. ferrugineipennis*, *Limonius agonus* (Say), *L. infuscatus*, *L. californicus*, *L*. *canus*, *S. a. destructor*, *H. bicolor*, *H. abbreviatus*, *A. haemorrhoidalis* and *H. glaucus*. Other taxa from the Coleopteran families, Elateridae, Carabidae, Scarabidae and Silphidae, confirmed the specificity of the assay. These novel primers can be used on elaterid populations included in this study, with caution taken for populations not tested in this study including locations outside of Canada. Applications of assays can include monitoring of native and invasive wireworm pests, risk assessments, and to aid in species‐specific studies for the further development of IPM strategies to combat these important agricultural pests. Future research should include testing the geographic scope of the designed primers outside of Canada and the development of PCR assays for pest elaterids not included in this study.

## CONFLICT OF INTEREST

The authors declare no conflict of interest.

## Supporting information


**Figure S1.** An example of primer specificity testing for the successful species‐specific primer assays of the 15 target species: (a) *Agriotes lineatus* (AL‐ND1‐292F and AL‐ND1‐582R, 312 bp), (b) *A. obscurus* (AO‐cytb‐123F and AO‐cytb‐353R, 256 bp), (c) *A. pubescens* (A‐lin/obs/pub‐F1 and A‐pub‐R2, 522 bp), (d) *A. sputator* (A‐spu‐F1 and A‐spu‐R2, 211 bp), (e) *A. ferrugineipennis* (Ferrug‐259F and Ferrug‐537R, 299 bp), (f) *A. mancus* (A‐man‐F2 and A‐man‐R1, 469 bp), (g) *Limonius canus* (Canus‐274F and Canus‐492R, 219 bp), (h) *L. infuscatus* (Infus‐245F and Infus‐572R, 300 bp), (i) *L. agonus* (Agonus‐222F and Agonus‐544R, 362 bp), (j) *L. californicus* (Cali‐189F and Cali‐492R, 310 bp), (k) *Hypnoidus abbreviatus* (HyA‐232F and HyA‐623R, 385 bp), (l) *H. bicolor* (HyB‐1F and HyB472R, 161 bp), (m) *Selatosomus aeripennis destructor* (SD‐124F and SD‐1R, 292 bp), (n) *Hadromorphus glaucus* (HG‐484F and HG‐23R, 463 bp), and (o) *Athous haemorrhoidalis* (Ath‐224F and Ath‐19R, 205 bp). Agarose gels show target DNA in the first well with the target species code in bold, as well as select non‐target DNA of click beetle species from the major agricultural areas of Canada (Table 1) that coexist with the target species.

**Figure S2.** Haplotype accumulation curves (a) and haplotype frequency bar plots (b) for the following target species: (1) *Agriotes obscurus*, (2) *A. sputator*, (3) *A. pubescens*, (4) *A. ferrugineipennis*, (5) *Limonius agonus*, (6) *L. canus*, (7) *L. infuscatus*, (8) *L. californicus*, (9) *Hypnoidus bicolor*, (10) *Selatosomus aeripennis destructor* and (11) *Athous haemorrhoidalis*. Sequences from our study were included, as well as sequences that our designed primers bind to from the NCBI database. In (a) the grey shaded area represents a 95% confidence interval, the dashed line represents the observed percentage of haplotype variation recovered for specimens sampled and the dotted line represents the estimated number of haplotypes to reach 95% haplotype variation recovery.

**Figure S3.** Median joining haplotype network showing (a) *Agriotes obscurus*, (b) *A. sputator*, (c) *A. pubescens*, (d) *A. ferrugineipennis*, (e) *Limonius agonus*, (f) *L. canus*, (g) *L. infuscatus*, (h) *L. californicus*, (i) *Hypnoidus bicolor*, (j) *Selatosomus aeripennis destructor* and (k) *Athous haemorrhoidalis*. Haplotypes found with more than one individual are bolded. The scale of the haplotype frequency is depicted by the size of the semi‐circle, and the colour corresponds to the geographic population of the collected individuals. Each hash‐mark represents a single‐nucleotide polymorphism (SNP). CYTB sequences from this study were used for *A. obscurus*, and COXI sequences from this study and sequences from the NCBI database that our designed primers bind to were used for the additional 10 species networks.

**Figure S4.** Samples tested across a panel of target and non‐target species from multiple geographic locations showing performance for each primer assay: (a) *Agriotes lineatus*, (b) *A. obscurus*, (c) *A. sputator*, (d) *A. mancus*, (e) *A. pubescens*, (f) *A. ferrugineipennis*, (g) *Limonius agonus*, (h) *L. infuscatus*, (i) *L. californicus*, (j) *L.canus*, (k) *Selatosomus aeripennis destructor*, (l) *Hypnoidus bicolor*, (m) *H. abbreviatus*, (n) *Hadromorphus glaucus* and (o) *Athous haemorrhoidalis*. Box colour represents amplification type, with grey as a true negative (TN), green a true positive (TP) and red a false negative (FN). Zero false positives (FP) were found during testing. The text colour for a species and its corresponding collection site is black for Canadian populations and blue for populations from the USA. Blocks of insect genus or family are annotated on the testing panel. False negatives were only found for (i) *L. californicus* and (h) *L. infuscatus* primer assays for populations tested in the USA.

**Table S1.** List of total target and non‐target species used in primer development and species‐specificity testing. Columns present the species name, collection sites, associated coordinates, number of DNA extracts used in study and number of sequences for each gene region.

**Table S2.** Intraspecific variation for each sequenced gene region per target species used in primer design. Intraspecific variation for those species with only one sequence per gene region are noted with an asterisk (*) and no sequence is indicated by a dash (–).

**Table S3.** Primer pair candidates composed of 99 primer candidates tested for specificity. Columns present the target species, primer names, candidate primer sequences (F and R denote forward and reverse, respectively) and amplicon size (bps).

**Table S4.** List of sequences from the Barcode of Life Database (BOLD) that were included in addition to the sequences from our study to use in the development of primers, haplotype accumulation curves and haplotype networks.

## Data Availability

The sequences generated from this research are available in BOLD Systems.
