# Supplementary material for: PCR‐based species identification tools for wireworms (Coleoptera: Elateridae) of economic importance in Canada
Source: Pest Manag Sci. 2026 Jun 25;82(10):9998–10010. doi: 10.1002/ps.71036 (PMC13569292; doi:10.1002/ps.71036)
Supplement: Supplementary file 1 [file PS-82-9998-s001.docx]

**Table S2** - Intraspecific variation for each sequenced gene region per target species used in primer design. Intraspecific variation for those species with only one sequence per gene region are noted with an asterisk (*), and no sequence is indicated by a dash (-).

| **Target Species** | **COXI** | **16S** | **12S** | **28S** | **18S** | **ITS2** | **CYTB** | **EF1** | **ATP6/8** | **ND1** | **ND2** | **ND3** | **ND4** | **ND5** | **ND6** |  |
| --- | --- | --- | --- | --- | --- | --- | --- | --- | --- | --- | --- | --- | --- | --- | --- | --- |
| *Agriotes lineatus* (Linnaeus) | 0.03 | * | * | * | * | 1.4 | 0 | 0.3 | * | 0 | * | * | 0 | 0.6 | * |  |
| *Agriotes obscurus* (Linnaeus) | 0.6 | - | - | - | * | 0.9 | 0.6 | 0.8 | * | 1.3 | * | * | 0.6 | 1.3 | - |  |
| *Agriotes sputator* (Linnaeus) | 0.1 | * | * | * | * | 0.3 | 0.2 | 2.7 | * | 0 | * | * | 1.3 | 0.8 | * |  |
| *Agriotes mancus* (Say) | 0.2 | - | - | * | * | 0.2 | 0.1 | 0.8 | * | * | - | * | 0.4 | 0.03 | * |  |
| *Agriotes pubescens* (Melsheimer) | 2.4 | - | * | * | * | * | 0 | 0.7 | * | 0.1 | * | * | 0.2 | 0.1 | * |  |
| *Agriotes ferrugineipennis* (LeConte) | 0.5 | - | * | - | * | 0.9 | 0.1 | 0.8 | * | 0 | * | * | 1 | * | * |  |
| *Limonius agonus* (Say) | 0.5 | * | * | * | * | 0.1 | 2.1 | 0.4 | * | 0.9 | - | * | 1.4 | 1.2 | - |  |
| *Limonius infuscatus* (Motschulsky) | 1.1 | * | * | * | * | 0.8 | 1.5 | 0.1 | * | 0 | * | * | - | - | * |  |
| *Limonius californicus* (Mannerheim) | 4 | * | * | * | * | 0 | 4.3 | - | * | 2.7 | * | * | 3.3 | 3.4 | - |  |
| *Limonius canus* (LeConte) | 0.6 | * | - | * | * | 0.8 | 0 | - | - | 0.1 | * | * | 0 | 0.2 | - |  |
| *Selatosomus aeripennis destructor* (Brown) | 0.7 | * | * | * | * | 0.9 | 0 | - | * | 0 | - | * | 0 | 0.7 | * |  |
| *Hypnoidus bicolor* (Eschscholtz) | 5.1 | - | - | - | - | - | - | - | - | - | - | - | - | - | - |  |
| *Hypnoidus abbreviatus* (Say) | 0.2 | - | - | - | - | - | - | - | - | - | - | - | - | - | - |  |
| *Athous haemorrhoidalis* (Fabricius) | 0.4 | - | - | - | - | - | - | - | - | - | - | - | - | - | - |  |
| *Hadromorphus glaucus* (Germar) | 0 | - | - | - | - | - | - | - | - | - | - | - | - | - | - |  |
