# Supplementary material for: PCR‐based species identification tools for wireworms (Coleoptera: Elateridae) of economic importance in Canada
Source: Pest Manag Sci. 2026 Jun 25;82(10):9998–10010. doi: 10.1002/ps.71036 (PMC13569292; doi:10.1002/ps.71036)
Supplement: Supplementary file 2 [file PS-82-9998-s002.docx]

**Table S4** - List of sequences from the Barcode of Life Database (BOLD) that were included in addition to the sequences from our study to use in the development of primers, haplotype accumulation curves and haplotype networks.

| **NCBI Accession Number** | **Location of origin** | **Species** |
| --- | --- | --- |
| HM542045 | Bad Kreuznach, Germany | *Agriotes sputator* |
| HM542046 | Cambridge, United Kingdom | *Agriotes sputator* |
| HM542047 | Orahovica, Croatia | *Agriotes sputator* |
| HM542048 | Bonn, Germany | *Agriotes sputator* |
| HM542049 | Bonn, Germany | *Agriotes sputator* |
| HM542050 | Niederoesterreich, Austria | *Agriotes sputator* |
| HM542051 | Niederoesterreich, Austria | *Agriotes sputator* |
| HM542052 | France | *Agriotes sputator* |
| KJ966203 | Mysinge, Sweden | *Agriotes sputator* |
| KJ966249 | Moeckelmossen, Sweden | *Agriotes sputator* |
| KM441588 | Nordrhein, Germany | *Agriotes sputator* |
| KM443912 | Bad Kreuznach, Germany | *Agriotes sputator* |
| KM444467 | Zwickau, Germany | *Agriotes sputator* |
| KM445546 | Bavaria, Germany | *Agriotes sputator* |
| KM447338 | Germersheim, Germany | *Agriotes sputator* |
| KM447480 | Jena, Germany | *Agriotes sputator* |
| KM448706 | Plaidt, Germany | *Agriotes sputator* |
| KM449245 | Swisttal, Germany | *Agriotes sputator* |
| KM450613 | Wesel, Germany | *Agriotes sputator* |
| KM451005 | Wesel, Germany | *Agriotes sputator* |
| KM451068 | Germersheim, Germany | *Agriotes sputator* |
| KM452476 | Germersheim, Germany | *Agriotes sputator* |
| KR124576 | Prince Edward Island, Canada | *Agriotes sputator* |
| KU906971 | Halberstadt, Germany | *Agriotes sputator* |
| KU911591 | Halberstadt, Germany | *Agriotes sputator* |
| KU912079 | Ichtershausen, Germany | *Agriotes sputator* |
| KU912280 | Pfalz, Germany | *Agriotes sputator* |
| KU912396 | Brandenburg, Germany | *Agriotes sputator* |
| KU912510 | Ichtershausen, Germany | *Agriotes sputator* |
| KU916976 | Ichtershausen, Germany | *Agriotes sputator* |
| MG055886 | Berwick, Nova Scotia, Canada | *Agriotes sputator* |
| MH159046 | Canada | *Agriotes sputator* |
| MG054638 | Peterborough, Ontario, Canada | *Agriotes mancus* |
| MG058154 | Peterborough, Ontario, Canada | *Agriotes mancus* |
| MG061795 | Guelph, Ontario, Canada | *Agriotes mancus* |
| MH118685 | Canada | *Agriotes mancus* |
| KR489859 | Grey County, Ontario, Canada | *Agriotes pubesens* |
| MG055864 | Hartington, Ontario, Canada | *Agriotes pubesens* |
| MG055032 | Clearwater, British Columbia, Canada | *Agriotes ferrugineipennis* |
| MG061015 | Clearwater, British Columbia, Canada | *Agriotes ferrugineipennis* |
| KF549839 | Hungary Horse, Montana, USA | *Limonius canus* |
| KF549840 | Grandview, Washington, USA | *Limonius canus* |
| KF549841 | Klickitat, Washington, USA | *Limonius canus* |
| KF549843 | Grandview, Washington, USA | *Limonius canus* |
| KF549844 | Grandview, Washington, USA | *Limonius canus* |
| KF549845 | Grandview, Washington, USA | *Limonius canus* |
| KF549859 | Wilber, Washington, USA | *Limonius infuscatus* |
| KF549861 | Bonners Ferry, Idaho, USA | *Limonius infuscatus* |
| KF549862 | Bonners Ferry, Idaho, USA | *Limonius infuscatus* |
| KF549863 | Davenport, Washington, USA | *Limonius infuscatus* |
| KF549864 | Nimrod Springs, Montana, USA | *Limonius infuscatus* |
| MT571677 | Spokane, Washington, USA | *Limonius infuscatus* |
| MT571678 | Garfield, Washington, USA | *Limonius infuscatus* |
| MT571693 | Adams City, Washington, USA | *Limonius infuscatus* |
| KF549757 | Conrad, Montana, USA | *Limonius californicus* |
| KF549758 | Conrad, Montana, USA | *Limonius californicus* |
| KF549759 | Conrad, Montana, USA | *Limonius californicus* |
| KF549760 | Davenport, Washington, USA | *Limonius californicus* |
| KF549770 | Rosalia, Washington, USA | *Limonius californicus* |
| KF549776 | Conrad, Montana, USA | *Limonius californicus* |
| KF549788 | Conrad, Montana, USA | *Limonius californicus* |
| KF549789 | Conrad, Montana, USA | *Limonius californicus* |
| KF549792 | Conrad, Montana, USA | *Limonius californicus* |
| KF549804 | Denton, Montana, USA | *Limonius californicus* |
| KF549805 | Denton, Montana, USA | *Limonius californicus* |
| KF549807 | Conrad, Montana, USA | *Limonius californicus* |
| KF549808 | Conrad, Montana, USA | *Limonius californicus* |
| KF549809 | Conrad, Montana, USA | *Limonius californicus* |
| KF549810 | Conrad, Montana, USA | *Limonius californicus* |
| KF549811 | Conrad, Montana, USA | *Limonius californicus* |
| KF549812 | Conrad, Montana, USA | *Limonius californicus* |
| KF549813 | Conrad, Montana, USA | *Limonius californicus* |
| KF549814 | Conrad, Montana, USA | *Limonius californicus* |
| KF549817 | Kalispell, Montana, USA | *Limonius californicus* |
| KF549818 | Kalispell, Montana, USA | *Limonius californicus* |
| KF549819 | Kalispell, Montana, USA | *Limonius californicus* |
| KF549820 | Kalispell, Montana, USA | *Limonius californicus* |
| KF549821 | Kalispell, Montana, USA | *Limonius californicus* |
| KF549825 | Conrad, Montana, USA | *Limonius californicus* |
| KF549826 | Conrad, Montana, USA | *Limonius californicus* |
| KF549829 | Conrad, Montana, USA | *Limonius californicus* |
| KF549830 | Conrad, Montana, USA | *Limonius californicus* |
| KM849992 | Saskatchewan, Canada | *Selatosomus aeripennis destructor* |
| HQ928215 | Canada | *Hypnoidus bicolor* |
| HQ928240 | Canada | *Hypnoidus bicolor* |
| JF888178 | Canada | *Hypnoidus bicolor* |
| JF888180 | Canada | *Hypnoidus bicolor* |
| JF888182 | Canada | *Hypnoidus bicolor* |
| JF888197 | Canada | *Hypnoidus bicolor* |
| JF888293 | Canada | *Hypnoidus bicolor* |
| JN266630 | Canada | *Hypnoidus bicolor* |
| KF549713 | Conrad, Montana, USA | *Hypnoidus bicolor* |
| KF549714 | Conrad, Montana, USA | *Hypnoidus bicolor* |
| KF549715 | Conrad, Montana, USA | *Hypnoidus bicolor* |
| KF549716 | Conrad, Montana, USA | *Hypnoidus bicolor* |
| KF549717 | Conrad, Montana, USA | *Hypnoidus bicolor* |
| KF549718 | Conrad, Montana, USA | *Hypnoidus bicolor* |
| KF549720 | Townsend, Montana, USA | *Hypnoidus bicolor* |
| KF549721 | Townsend, Montana, USA | *Hypnoidus bicolor* |
| KF549722 | Townsend, Montana, USA | *Hypnoidus bicolor* |
| KF549723 | Townsend, Montana, USA | *Hypnoidus bicolor* |
| KF549724 | Townsend, Montana, USA | *Hypnoidus bicolor* |
| KF549725 | Townsend, Montana, USA | *Hypnoidus bicolor* |
| KF549727 | Townsend, Montana, USA | *Hypnoidus bicolor* |
| KF549728 | Townsend, Montana, USA | *Hypnoidus bicolor* |
| KF549729 | Conrad, Montana, USA | *Hypnoidus bicolor* |
| KF549730 | Conrad, Montana, USA | *Hypnoidus bicolor* |
| KF549731 | Conrad, Montana, USA | *Hypnoidus bicolor* |
| KF549732 | Conrad, Montana, USA | *Hypnoidus bicolor* |
| KF549733 | Conrad, Montana, USA | *Hypnoidus bicolor* |
| KF549734 | Conrad, Montana, USA | *Hypnoidus bicolor* |
| KF549735 | Conrad, Montana, USA | *Hypnoidus bicolor* |
| KF549736 | Conrad, Montana, USA | *Hypnoidus bicolor* |
| KF549737 | Conrad, Montana, USA | *Hypnoidus bicolor* |
| KF549738 | Conrad, Montana, USA | *Hypnoidus bicolor* |
| KF549739 | Conrad, Montana, USA | *Hypnoidus bicolor* |
| KF549740 | Conrad, Montana, USA | *Hypnoidus bicolor* |
| KF549741 | Conrad, Montana, USA | *Hypnoidus bicolor* |
| KF549742 | Conrad, Montana, USA | *Hypnoidus bicolor* |
| KF549743 | Conrad, Montana, USA | *Hypnoidus bicolor* |
| KF549744 | Conrad, Montana, USA | *Hypnoidus bicolor* |
| KF549745 | Conrad, Montana, USA | *Hypnoidus bicolor* |
| KF549746 | Idaho, USA | *Hypnoidus bicolor* |
| KF549748 | Montana, USA | *Hypnoidus bicolor* |
| KJ202736 | Churchill, Manitoba, Canada | *Hypnoidus bicolor* |
| KJ202807 | Churchill, Manitoba, Canada | *Hypnoidus bicolor* |
| KJ203171 | Churchill, Manitoba, Canada | *Hypnoidus bicolor* |
| KJ203182 | Churchill, Manitoba, Canada | *Hypnoidus bicolor* |
| KJ203208 | Churchill, Manitoba, Canada | *Hypnoidus bicolor* |
| KJ203540 | Churchill, Manitoba, Canada | *Hypnoidus bicolor* |
| KJ203867 | Churchill, Manitoba, Canada | *Hypnoidus bicolor* |
| KJ203931 | Churchill, Manitoba, Canada | *Hypnoidus bicolor* |
| KM841558 | Banff, Alberta, Canada | *Hypnoidus bicolor* |
| KM841855 | Banff, Alberta, Canada | *Hypnoidus bicolor* |
| KM845259 | Banff, Alberta, Canada | *Hypnoidus bicolor* |
| KR915686 | Yoho National Park, British Columbia, Canada | *Hypnoidus bicolor* |
| KU875259 | Alaska, USA | *Hypnoidus bicolor* |
| KU875260 | Fairbanks, Alaska, USA | *Hypnoidus bicolor* |
| MF632432 | Glacier National Park, British Columbia, Canada | *Hypnoidus bicolor* |
| MF632825 | Glacier National Park, British Columbia, Canada | *Hypnoidus bicolor* |
| MF633017 | Glacier National Park, British Columbia, Canada | *Hypnoidus bicolor* |
| MF633559 | Glacier National Park, British Columbia, Canada | *Hypnoidus bicolor* |
| MF633640 | Glacier National Park, British Columbia, Canada | *Hypnoidus bicolor* |
| MF634973 | Glacier National Park, British Columbia, Canada | *Hypnoidus bicolor* |
| MF636381 | Glacier National Park, British Columbia, Canada | *Hypnoidus bicolor* |
| MF637556 | Glacier National Park, British Columbia, Canada | *Hypnoidus bicolor* |
| MF638849 | Glacier National Park, British Columbia, Canada | *Hypnoidus bicolor* |
| MF639916 | Glacier National Park, British Columbia, Canada | *Hypnoidus bicolor* |
| MF640613 | Glacier National Park, British Columbia, Canada | *Hypnoidus bicolor* |
| MF640810 | Glacier National Park, British Columbia, Canada | *Hypnoidus bicolor* |
| MF642273 | Glacier National Park, British Columbia, Canada | *Hypnoidus bicolor* |
| MG054694 | British Columbia, Canada | *Hypnoidus bicolor* |
| MG055162 | British Columbia, Canada | *Hypnoidus bicolor* |
| MG058427 | Winnipeg, Manitoba, Canada | *Hypnoidus bicolor* |
| MG059482 | British Columbia, Canada | *Hypnoidus bicolor* |
| MH118680 | Canada | *Hypnoidus abbreviatus* |
| MH169715 | Canada | *Hypnoidus abbreviatus* |
| KM841522 | Alberta, Canada | *Hadromorphus glaucus* |
| KM842278 | Alberta, Canada | *Hadromorphus glaucus* |
| KM845552 | Alberta, Canada | *Hadromorphus glaucus* |
| AJ862812 |  | *Athous haemorrhoidalis* |
| DQ198570 | United Kingdom | *Athous haemorrhoidalis* |
| HQ953319 | Germany | *Athous haemorrhoidalis* |
| KJ962837 | Finland | *Athous haemorrhoidalis* |
| KJ963733 | Borgholm, Sweden | *Athous haemorrhoidalis* |
| KJ964517 | Sweden | *Athous haemorrhoidalis* |
| KJ965006 | Sweden | *Athous haemorrhoidalis* |
| KJ965771 | Aland Islands, Finland | *Athous haemorrhoidalis* |
| KJ966403 | Aland Islands, Finland | *Athous haemorrhoidalis* |
| KJ966761 | Finland | *Athous haemorrhoidalis* |
| KM285904 | France | *Athous haemorrhoidalis* |
| KM440508 | Niedercrinitz, Germany | *Athous haemorrhoidalis* |
| KM440586 | Erfstadt-Bliesheim, Germany | *Athous haemorrhoidalis* |
| KM440683 | Dachau, Germany | *Athous haemorrhoidalis* |
| KM441613 | Bornheim-Hemmerich, Germany | *Athous haemorrhoidalis* |
| KM444800 | Germersheim, Germany | *Athous haemorrhoidalis* |
| KM447860 | Schleiden-Wolfgarten, Germany | *Athous haemorrhoidalis* |
| KM448068 | Moselkern, Germany | *Athous haemorrhoidalis* |
| KM450593 | Rhone-Alpes, France | *Athous haemorrhoidalis* |
| KM451077 | Schwaben, Germany | *Athous haemorrhoidalis* |
| KM452422 | Erfstadt-Bliesheim, Germany | *Athous haemorrhoidalis* |
| KU906228 | Altrip, Germany | *Athous haemorrhoidalis* |
| KU907005 | Germany | *Athous haemorrhoidalis* |
| KU907754 | Brandenburg, Germany | *Athous haemorrhoidalis* |
| KU909227 | Saxonyburg, Germany | *Athous haemorrhoidalis* |
| KU911239 | Mocklenburg-Vorpommern, Germany | *Athous haemorrhoidalis* |
| KU911260 | Saxony, Germany | *Athous haemorrhoidalis* |
| KU912561 | Saxony, Germany | *Athous haemorrhoidalis* |
| KU913101 | Saxony, Germany | *Athous haemorrhoidalis* |
| KU914500 | Altrip, Germany | *Athous haemorrhoidalis* |
| KU914848 | Saxonyburg, Germany | *Athous haemorrhoidalis* |
| KU916923 | Saxony-Anhalt, Germany | *Athous haemorrhoidalis* |
| KU916992 | Macklenburg-Vorpommern, Germany | *Athous haemorrhoidalis* |
| KU917744 | Morsbach, Germany | *Athous haemorrhoidalis* |
| KU917881 | Saxonyburg, Germany | *Athous haemorrhoidalis* |
| KU919057 | Saxonyburg, Germany | *Athous haemorrhoidalis* |
| KU919504 | Altrip, Germany | *Athous haemorrhoidalis* |
| MG057204 | Cambridge, Ontario, Canada | *Athous haemorrhoidalis* |
| MW259700 | Germany | *Athous haemorrhoidalis* |
| MW259876 | Germany | *Athous haemorrhoidalis* |
