# Supplementary material for: PCR‐based species identification tools for wireworms (Coleoptera: Elateridae) of economic importance in Canada
Source: Pest Manag Sci. 2026 Jun 25;82(10):9998–10010. doi: 10.1002/ps.71036 (PMC13569292; doi:10.1002/ps.71036)
Supplement: Supplementary file 3 [file PS-82-9998-s003.docx]

**Supplementary Figure 2 –** Haplotype accumulation curves (a) and haplotype frequency bar plots (b) for the following target species: (1) *Agriotes obscurus*, (2) *A. sputator* (3) *A. pubescens*, (4) *A. ferrugineipennis*, (5) *Limonius agonus*, (6) *L. canus*, (7) *L. infuscatus*, (8) *L. californicus*, (9) *Hypnoidus bicolor*, (10) *Selatosomus aeripennis destructor* and (11) *Athous haemorrhoidalis*. Sequences from our study were included, as well as sequences that our designed primers bind to from the NCBI database. In (a) the grey shaded area represents a 95% confidence interval, the dashed line represents the observed percentage of haplotype variation recovered for specimens sampled and the dotted line represents the estimated number of haplotypes to reach 95% haplotype variation recovery.
