# Supplementary material for: PCR‐based species identification tools for wireworms (Coleoptera: Elateridae) of economic importance in Canada
Source: Pest Manag Sci. 2026 Jun 25;82(10):9998–10010. doi: 10.1002/ps.71036 (PMC13569292; doi:10.1002/ps.71036)
Supplement: Supplementary file 4 [file PS-82-9998-s004.docx]

**Supplementary Figure 3 –** Median joining haplotype network showing: (a) *Agriotes obscurus*, (b) *Agriotes sputator,* (c) *Agriotes pubescens*, (d) *Agriotes ferrugineipennis*, (e) *Limonius agonus*, (f) *Limonius canus*, (g) *Limonius infuscatus*, (h) *Limonius californicus*, (i) *Hypnoidus bicolor*, (j) *Selatosomus aeripennis destructor* and (k) *Athous haemorrhoidalis*. Haplotypes found with more than one individual are bolded. The scale of the haplotype frequency is depicted by the size of the semi-circle, and the colour corresponds to the geographic population of the collected individuals. Each hash-mark represents a single-nucleotide polymorphism (SNP). CYTB sequences from this study were used for *A. obscurus*, and COXI sequences from this study and sequences from the NCBI database that our designed primers bind to were used for the additional 10 species networks.
