# Supplementary material for: PCR‐based species identification tools for wireworms (Coleoptera: Elateridae) of economic importance in Canada
Source: Pest Manag Sci. 2026 Jun 25;82(10):9998–10010. doi: 10.1002/ps.71036 (PMC13569292; doi:10.1002/ps.71036)
Supplement: Supplementary file 5 [file PS-82-9998-s005.docx]

**Table S3** - Primer pair candidates composed of 99 primer candidates tested for specificity. Columns present the target species, primer names, candidate primer sequences (F and R denote forward and reverse, respectively) and amplicon size (bps).

| **Target Species** | **Gene Region** | **Primer Name** | **Candidate Primer Sequences (5' to 3')** | **Amplicon Size (bps)** |
| --- | --- | --- | --- | --- |
| *Agriotes lineatus* (Linnaeus) | COXI | A-lin/obs/pub-F1 | CCCTGGATCACTAATCGGAAAT | 440 |
|  |  | A-lin-R1 | TTGCTACTGCTCAAACGAATAAAG |  |
|  | COXI | A-lin/obs/pub-F1 | listed above | 508 |
|  |  | A-lin-R2 | GTCAGTTAATAGTATTGTAATTGCTCCAG |  |
|  | COXI | A-lin/obs/pub-F1 | listed above | 403 |
|  |  | A-lin-R3 | TGTAATTCCTGTTGATCGCATA |  |
|  | ND1 | AL-ND1-292F | TTTGCTTTAGTTTTATGGGTTTGTGTA | 321 |
|  |  | AL-ND1-594R | AACAAACCATACCATCCCG |  |
|  | ND1 | AL-ND1-292F | listed above | 312 |
|  |  | AL-ND1-582R | TACCATCCCGAGAGGAATACTC |  |
|  | ND1 | AL-ND1-292F | listed above | 213 |
|  |  | AL-ND1-477R | CAATAAAATTAAAGCTAAACTTACCTCA |  |
|  | ND1 | AL-ND1-144F | TCAAATTCGTAAAGGGCCCAATAAG | 469 |
|  |  | AL-ND1-594R | listed above |  |
|  | ND1 | AL-ND1-144F | listed above | 460 |
|  |  | AL-ND1-582R | listed above |  |
|  | ND1 | AL-ND1-144F | listed above | 361 |
|  |  | AL-ND1-477R | listed above |  |
|  |  |  |  |  |
| *Agriotes obscurus* (Linnaeus) | COXI | A-lin/obs/pub-F1 | listed above | 439 |
|  |  | A-obs-R1 | TGCCACTGCTCAAACAAATAAA |  |
|  | COXI | A-lin/obs/pub-F1 | listed above | 483 |
|  |  | A-obs-R2 | CCTGCTAAGACAGGTAAGGATAAA |  |
|  | COXI | A-lin/obs/pub-F1 | listed above | 455 |
|  |  | A-obs-R3 | GTAGTAGTGCAGTGATTGCCACTG |  |
|  | CYTB | AO-cytb-101F | AACCTTAACCCGGTTCTTT | 202 |
|  |  | AO-cytb-278R | AGTTAAGATTATTAATGTTACATTG |  |
|  | CYTB | AO-cytb-101F | listed above | 274 |
|  |  | AO-cytb-353R | AGTTACTAAAGGATTGGCAGGA |  |
|  | CYTB | AO-cytb-123F | TACACTACACTTTTTACTACCATTTG | 256 |
|  |  | AO-cytb-353R | listed above |  |
|  |  |  |  |  |
| *Agriotes sputator* (Linnaeus) | COXI | A-spu-F1 | CCCTGGGTCATTAATCGGAAAT | 516 |
|  |  | A-spu-R1 | AGATTACGGTCTGTTAGTAACATAG |  |
|  | COXI | A-spu-F1 | listed above | 211 |
|  |  | A-spu-R2 | TAATAGAAGAGACAATGAAGGTGGAAGA |  |
|  | COXI | A-spu-F1 | listed above | 449 |
|  |  | A-spu-R3 | AAGCTGTGATTGCTACTGCTCATA |  |
|  |  |  |  |  |
| *Agriotes mancus* (Say) | COXI | A-man-F1 | CGGATCATTAATCGGAAATGACCAGATCTAC | 467 |
|  |  | A-man-R1 | GTAAGGATAGGAGGAGGAGTAGG |  |
|  | COXI | A-man-F1 | listed above | 337 |
|  |  | A-man-R2 | TGAAATTCCTGCCAGGTGTAA |  |
|  | COXI | A-man-F2 | CCCGGATCATTAATCGGAAAT | 469 |
|  |  | A-man-R1 | listed above |  |
|  | COXI | A-man-F2 | listed above | 339 |
|  |  | A-man-R2 | listed above |  |
|  |  |  |  |  |
| *Agriotes pubescens* (Melsheimer) | COXI | A-lin/obs/pub-F1 | listed above | 224 |
|  |  | A-pub-R1 | CAATTCTTCTTATTAGAAGGAGGGATA |  |
|  | COXI | A-lin/obs/pub-F1 | listed above | 522 |
|  |  | A-pub-R2 | GTGTTTAAATTTCGATCAGTAAGTA |  |
|  | COXI | A-lin/obs/pub-F1 | listed above | 254 |
|  |  | A-pub-R3 | CAGTTCACCCTGTTCCTGC |  |
|  |  |  |  |  |
| *Agriotes ferrugineipennis* (LeConte) | COXI | Ferrug-201F | TTGATTAGTACCACTAATGCTAGGTG | 361 |
|  |  | Ferrug-537R | TGGAAGTGATAGGAGTAGAAGTAGG |  |
|  | COXI | Ferrug-201F | listed above | 398 |
|  |  | Ferrug-575R | GAAGTAGGGATAATGAAGGGGGTA |  |
|  | COXI | Ferrug-201F | listed above | 262 |
|  |  | Ferrug-462R | TGTTGAGATGAAGTTTACGGCG |  |
|  | COXI | Ferrug-275F | TACCCCCTTCATTATCCCTACTTC | 287 |
|  |  | Ferrug-537R | listed above |  |
|  | COXI | Ferrug-275F | listed above | 324 |
|  |  | Ferrug-575R | listed above |  |
|  | COXI | Ferrug-275F | listed above | 188 |
|  |  | Ferrug-462R | listed above |  |
|  | COXI | Ferrug-259F | CATAAGATTCTGGTTTCTACCCCCT | 304 |
|  |  | Ferrug-537R | listed above |  |
|  | COXI | Ferrug-259F | listed above | 341 |
|  |  | Ferrug-575R | listed above |  |
|  | COXI | Ferrug-259F | listed above | 205 |
|  |  | Ferrug-462R | listed above |  |
|  |  |  |  |  |
| *Limonius agonus* (Say) | COXI | Agonus-43F | AATTCGTGCAGAACTCGGAAAC | 182 |
|  |  | Agonus-230R | TACGAGGAAAGGCTATGTCAG |  |
|  | COXI | Agonus-43F | listed above | 358 |
|  |  | Agonus-407R | GATTCCTGCCAAGTGTAGACTA |  |
|  | COXI | Agonus-43F | listed above | 503 |
|  |  | Agonus-544R | CTGCAAGAACTGGTAGAGAG |  |
|  | COXI | Agonus-43F | listed above | 487 |
|  |  | Agonus-555R | AGAGAGGAGAAGAAGGAGAGC |  |
|  | COXI | Agonus-58F | AGAACTCGGAAACCCTGGTTCG | 349 |
|  |  | Agonus-407R | listed above |  |
|  | COXI | Agonus-58F | listed above | 477 |
|  |  | Agonus-555R | listed above |  |
|  | COXI | Agonus-196F | AGCCCCTGACATAGCCTTTC | 202 |
|  |  | Agonus-407R | listed above |  |
|  | COXI | Agonus-196F | listed above | 347 |
|  |  | Agonus-544R | listed above |  |
|  | COXI | Agonus-196F | listed above | 331 |
|  |  | Agonus-555R | listed above |  |
|  | COXI | Agonus-222F | TTGACTAGTACCACTTATACTAGGA | 371 |
|  |  | Agonus-544R | listed above |  |
|  | COXI | Agonus-254F | GCCATCTCTTTCCCTTCTTTA | 293 |
|  |  | Agonus-544R | listed above |  |
|  |  |  |  |  |
| *Limonius infuscatus* (Motschulsky) | COXI | Infuscatus-204F | GCTTATGCTAGGAGCCCCA | 250 |
|  |  | Infuscatus-441R | GGTGGAGATAAAGTTTACTGCT |  |
|  | COXI | Infuscatus-204F | listed above | 347 |
|  |  | Infuscatus-531R | GTAAGGAGAGAAGTAATAGTAGAGCTGTA |  |
|  | COXI | Infuscatus-204F | listed above | 419 |
|  |  | Infuscatus-609R | CTGCGGGATCAAAAAATGAGGTA |  |
|  | COXI | Infuscatus-204F | listed above | 191 |
|  |  | Infuscatus-403R | GAAAATTGCCAAATCAACGGAGGAT |  |
|  | COXI | Infuscatus-204F | listed above | 360 |
|  |  | Infuscatus-572R | CCGGCAAGAACTGGTAAGG |  |
|  | COXI | Infuscatus-245F | CCTTCCTCCATCGCTTTCC | 190 |
|  |  | Infuscatus-441R | listed above |  |
|  | COXI | Infuscatus-245F | listed above | 287 |
|  |  | Infuscatus-531R | listed above |  |
|  | COXI | Infuscatus-245F | listed above | 359 |
|  |  | Infuscatus-609R | listed above |  |
|  | COXI | Infuscatus-245F | listed above | 300 |
|  |  | Infuscatus-572R | listed above |  |
|  | COXI | Infuscatus-267F | CTGATTCCTTCCTCCATCGC | 196 |
|  |  | Infuscatus-441R | listed above |  |
|  | COXI | Infuscatus-267F | listed above | 306 |
|  |  | Infuscatus-572R | listed above |  |
|  | COXI | Infuscatus-267F | listed above | 293 |
|  |  | Infuscatus-531R | listed above |  |
|  | COXI | Infuscatus-267F | listed above | 365 |
|  |  | Infuscatus-609R | listed above |  |
|  | COXI | Infuscatus-342F | TGTTTATCCACCCTTATCAGCAAACATC | 218 |
|  |  | Infuscatus-531R | listed above |  |
|  | COXI | Infuscatus-245F | listed above | 287 |
|  |  | Infuscatus-531R | listed above |  |
|  | COXI | Infuscatus-245F | listed above | 359 |
|  |  | Infuscatus-609R | listed above |  |
|  | COXI | Infuscatus-245F | listed above | 300 |
|  |  | Infuscatus-572R | listed above |  |
|  | COXI | Infuscatus-267F | CTGATTCCTTCCTCCATCGC | 196 |
|  |  | Infuscatus-441R | listed above |  |
|  | COXI | Infuscatus-267F | listed above | 306 |
|  |  | Infuscatus-572R | listed above |  |
|  | COXI | Infuscatus-267F | listed above | 293 |
|  |  | Infuscatus-531R | listed above |  |
|  | COXI | Infuscatus-267F | listed above | 365 |
|  |  | Infuscatus-609R | listed above |  |
|  | COXI | Infuscatus-342F | TGTTTATCCACCCTTATCAGCAAACATC | 218 |
|  |  | Infuscatus-531R | listed above |  |
|  | COXI | Infuscatus-342F | listed above | 290 |
|  |  | Infuscatus-609R | listed above |  |
|  | COXI | Infuscatus-342F | listed above | 231 |
|  |  | Infuscatus-572R | listed above |  |
|  |  |  |  |  |
| *Limonius californicus* (Mannerheim) | COXI | Californicus-189F | GTTCGGAAACTGACTAGTTCCA | 310 |
|  |  | Californicus-492R | ACGGTCGAAGGTAATTCCGGTA |  |
|  | COXI | Californicus-189F | listed above | 365 |
|  |  | Californicus-534R | GTGAAAGCAAGAGGAGAAGG |  |
|  | COXI | Californicus-273F | TTACCCCCGTCCCTTTCTCTT | 228 |
|  |  | Californicus-492R | listed above |  |
|  | COXI | Californicus-273F | listed above | 283 |
|  |  | Californicus-534R | listed above |  |
|  |  |  |  |  |
| *Limonius canus* (LeConte) | COXI | Canus-5F | TTGGAGCATGGCCGGAATAC | 355 |
|  |  | Canus-358R | CCCTCTGTGTGCAATGTTG |  |
|  | COXI | Canus-5F | listed above | 586 |
|  |  | Canus-585R | GGTGTTTAGATTACGATCTGTCAATAAT |  |
|  | COXI | Canus-5F | listed above | 544 |
|  |  | Canus-552R | TGCTAGGACTGGGAGAGAAAG |  |
|  | COXI | Canus-5F | listed above | 466 |
|  |  | Canus-492R | GGTGATTCCTGTGGATCGC |  |
|  | COXI | Canus-193-F | TTCGGAAACTGATTAGTGCCC | 189 |
|  |  | Canus-358R | listed above |  |
|  | COXI | Canus-193-F | listed above | 420 |
|  |  | Canus-585R | listed above |  |
|  | COXI | Canus-193-F | listed above | 378 |
|  |  | Canus-552R | listed above |  |
|  | COXI | Canus-193-F | listed above | 300 |
|  |  | Canus-492R | listed above |  |
|  | COXI | Canus-251F | CCTACCCCCGTCTCTTTCC | 298 |
|  |  | Canus-552R | listed above |  |
|  | COXI | Canus-251F | listed above | 340 |
|  |  | Canus-585R | listed above |  |
|  | COXI | Canus-251F | listed above | 220 |
|  |  | Canus-492R | listed above |  |
|  | COXI | Canus-274F | CTACCCCCGTCTCTTTCCCTC | 297 |
|  |  | Canus-552R | listed above |  |
|  | COXI | Canus-274F | listed above | 339 |
|  |  | Canus-585R | listed above |  |
|  | COXI | Canus-274F | listed above | 219 |
|  |  | Canus-492R | listed above |  |
|  |  |  |  |  |
| *Selatosomus aeripennis destructor* (Brown) | COXI | Destructor-42F | TCCCGGCTCTCTCATTGGTAAC | 469 |
|  |  | Destructor-537R | CAGAGATAAGAGTAGAAGGAGGG |  |
|  | COXI | Destructor-42F | listed above | 365 |
|  |  | Destructor-454R | TGAAATTTACAGCCCCCAGG |  |
|  | COXI | Destructor-82F | GTAATCGTAACAGCCCACGCC | 431 |
|  |  | Destructor-537R | listed above |  |
|  | COXI | Destructor-82F | listed above | 327 |
|  |  | Destructor-454R | listed above |  |
|  | COXI | Destructor-221F | CCTCCCACCCTCTCTATCA | 286 |
|  |  | Destructor-537R | listed above |  |
|  | COXI | Destructor-221F | listed above | 182 |
|  |  | Destructor-454R | listed above |  |
|  | COXI | Destructor-42F | listed above | 356 |
|  |  | Destructor-380R | CAGCCCCCAGGATTGATGAG |  |
|  | COXI | Destructor-82F | listed above | 318 |
|  |  | Destructor-380R | listed above |  |
|  | COXI | Destructor-74F | GAGCTGAACTAGGTAATCCCG | 372 |
|  |  | Destructor-380R | listed above |  |
|  | COXI | Destructor-74F | listed above | 381 |
|  |  | Destructor-454R | listed above |  |
|  | COXI | Destructor-74F | listed above | 485 |
|  |  | Destructor-537R | listed above |  |
|  | COXI | SD-1F | AAGATTCTGATTCCTCCCACCCT | 292 |
|  |  | SD-124R | TAAGAGTAGAAGGAGGGCTG |  |
|  | COXI | SD-1F | listed above | 274 |
|  |  | SD-142R | TGTGATAGCTACTGCCCATA |  |
|  |  |  |  |  |
| *Hypnoidus bicolo*r (Eschscholtz) | COXI | HyB-197F | GTAATTGATTAGTACCCCTCATAC | 236 |
|  |  | HyB-411R | AGACGAAATACCAGCTAGATGT |  |
|  | COXI | HyB-276F | ACCCCCTTCACTATCACTTCTA | 157 |
|  |  | HyB-411R | listed above |  |
|  | COXI | HyB-197F | listed above | 240 |
|  |  | HyB-415R | GGATAGACGAAATACCAGCTAG |  |
|  | COXI | HyB-276F | listed above | 161 |
|  |  | HyB-415R | listed above |  |
|  | COXI | HyB-197F | listed above | 248 |
|  |  | HyB-422R | TGCTCCTAGGATAGACGAAATAC |  |
|  | COXI | HyB-276F | listed above | 169 |
|  |  | HyB-422R | listed above |  |
|  | COXI | HyB-472F | TGGTAATTGATTAGTACCCCTCA | 161 |
|  |  | HyB-1R | AAGGAGGGTATACTGTTCAACC |  |
|  | COXI | HyB-521F | GTAATTGTAACAGCCCATGC | 229 |
|  |  | HyB-1R | listed above |  |
|  |  |  |  |  |
| *Hypnoidus abbreviatus* (Say) | COXI | HyA-273F | TCTACCCCCATCACTATCTC | 272 |
|  |  | HyA-521R | GAAGAAGAGCAGTAATTGCTACGG |  |
|  | COXI | HyA-336F | TGGATGAACAGTTTACCCTCCACTC | 212 |
|  |  | HyA-521R | listed above |  |
|  | COXI | HyA-336F | TGGATGAACAGTTTACCCTCCACTC | 212 |
|  |  | HyA-521R | listed above |  |
|  | COXI | HyA-273F | listed above | 283 |
|  |  | HyA-531R | TGAAAGGAGAAGAAGAAGAGCAGTA |  |
|  | COXI | HyA-336F | listed above | 523 |
|  |  | HyA-531R | listed above |  |
|  | COXI | HyA-623F | AGGAACATCCTTAAGTCTCCT | 385 |
|  |  | HyA-232R | AGAGGAGATTCCAGCTAAGTG |  |
|  | COXI | HyA-623F | listed above | 375 |
|  |  | HyA-241R | TCCAGCTAAGTGTAGACTGA |  |
|  | COXI | HyA-592F | ATCCTTAAGTCTCCTAATCCGTGCA | 379 |
|  |  | HyA-232R | listed above |  |
|  |  |  |  |  |
| *Hadromorphus glaucus* (Germar) | COXI | HG-484F | CCTGGGTCTCTTATTGGAAACG | 463 |
|  |  | HG-23R | ACAATAGGAGTAGTAGGGCAGTG |  |
|  | COXI | HG-484F | listed above | 207 |
|  |  | HG-282R | GAGAAGGGATAGAGAAGGAGGT |  |
|  |  |  |  |  |
| *Athous haemorrhoidalis* (Fabricius) | COXI | Ath-220F | TATCAGCCAATATTGCCCATAGAGG | 200 |
|  |  | Ath-19R | AGAGATAGGAGTAGGAGGAGTGCA |  |
|  | COXI | Ath-224F | ACCCTTATCAGCCAATATTGCCCAT | 205 |
|  |  | Ath-19R | listed above |  |
|  | COXI | Ath-220F | listed above | 196 |
|  |  | Ath-25R | AGGAGTAGGAGGAGTGCAG |  |
|  | COXI | Ath-224F | listed above | 201 |
|  |  | Ath-25R | listed above |  |
