# Supplementary material for: PCR‐based species identification tools for wireworms (Coleoptera: Elateridae) of economic importance in Canada
Source: Pest Manag Sci. 2026 Jun 25;82(10):9998–10010. doi: 10.1002/ps.71036 (PMC13569292; doi:10.1002/ps.71036)
Supplement: Supplementary file 6 [file PS-82-9998-s006.docx]

**Supplementary Figure 1 –** An example of primer specificity testing for the successful species-specific primer assays of the 15 target species: (a) *Agriotes lineatus* (AL-ND1-292F and AL-ND1-582R, 312 bp), (b) *A. obscurus* (AO-cytb-123F and AO-cytb-353R, 256 bp), (c) *A. pubescens* (A-lin/obs/pub-F1 and A-pub-R2, 522 bp), (d) *A. sputator* (A-spu-F1 and A-spu-R2, 211 bp), (e) *A. ferrugineipennis* (Ferrug-259F and Ferrug-537R, 299 bp), (f) *A. mancus* (A-man-F2 and A-man-R1, 469 bp), (g) *Limonius canus* (Canus-274F and Canus-492R, 219 bp), (h) *L. infuscatus* (Infus-245F and Infus-572R, 300 bp), (i) *L. agonus* (Agonus-222F and Agonus-544R, 362 bp), (j) *L. californicus* (Cali-189F and Cali-492R, 310 bp), (k) *Hypnoidus abbreviatus* (HyA-232F and HyA-623R, 385 bp), (l) *H. bicolor* (HyB-1F and HyB472R, 161 bp), (m) *Selatosomus aeripennis destructor* (SD-124F and SD-1R, 292 bp), (n) *Hadromorphus glaucus* (HG-484F and HG-23R, 463 bp)., and (o) *Athous haemorrhoidalis* (Ath-224F and Ath-19R, 205 bp). Agarose gels show target DNA in the first well with the target species code in bold, as well as select non-target DNA of click beetle species from the major agricultural areas of Canada (Table 1) that co-exist with the target species.
