# Supplementary material for: PCR‐based species identification tools for wireworms (Coleoptera: Elateridae) of economic importance in Canada
Source: Pest Manag Sci. 2026 Jun 25;82(10):9998–10010. doi: 10.1002/ps.71036 (PMC13569292; doi:10.1002/ps.71036)
Supplement: Supplementary file 7 [file PS-82-9998-s007.docx]

**Table S1 –** List of total target and non-target species used in primer development and species-specificity testing. Columns present the species name, collection sites, associated coordinates, number of DNA extracts used in study, and number of sequences for each gene region.

|  |  |  |  | **Number of Sequences** | | | | | | | | | | | | | | | | | |
| --- | --- | --- | --- | --- | --- | --- | --- | --- | --- | --- | --- | --- | --- | --- | --- | --- | --- | --- | --- | --- | --- |
| **Species** | **Collection Sites** | **Coordinates** | **DNA Extracts** | **COXI** | | **16S/12S** | | **28S** | **18S** | **ITS2** | **CYTB** | **EF1** | | **ATP6/8** | | **ND1** | **ND2** | **ND3** | **ND4** | **ND5** | **ND6** |
| **Target Species** |  |  |  |  |  | |  | |  |  |  | |  | |  |  |  |  |  |  |  |
| **Elateridae** |  |  |  |  |  | |  | |  |  |  | |  | |  |  |  |  |  |  |  |
| *Agriotes lineatus* (Linnaeus) | Agassiz, BC | 49.24336, -121.7588 | 6 | 4 | 2 | | 1 | | 1 | 4 | 3 | 3 | | 1 | | 3 | 1 | 1 | 3 | 3 | 1 |
|  |  |  |  |  |  | |  | |  |  |  | |  | |  |  |  |  |  |  |  |
|  | Creston, BC | 49.09215, -116.4692 | 5 |  |  | |  | |  |  |  | |  | |  |  |  |  |  |  |  |
|  |  |  |  |  |  | |  | |  |  |  | |  | |  |  |  |  |  |  |  |
|  | Rock Creek, BC | 49.12939, -118.9936 | 5 |  |  | |  | |  |  |  | |  | |  |  |  |  |  |  |  |
|  |  |  |  |  |  | |  | |  |  |  | |  | |  |  |  |  |  |  |  |
|  | Grand Forks, BC | 49.01263, -118.49425 | 4 |  |  | |  | |  |  |  | |  | |  |  |  |  |  |  |  |
|  |  |  |  |  |  | |  | |  |  |  | |  | |  |  |  |  |  |  |  |
|  | Revelstoke, BC | 50.98448, -118.22681 | 5 |  |  | |  | |  |  |  | |  | |  |  |  |  |  |  |  |
|  |  |  |  |  |  | |  | |  |  |  | |  | |  |  |  |  |  |  |  |
|  | Malakwa, BC | 50.95443, -118.76123 | 3 |  |  | |  | |  |  |  | |  | |  |  |  |  |  |  |  |
|  |  |  |  |  |  | |  | |  |  |  | |  | |  |  |  |  |  |  |  |
|  | Salmon Arm, BC | 50.67931, -119.21733 | 3 |  |  | |  | |  |  |  | |  | |  |  |  |  |  |  |  |
|  |  |  |  |  |  | |  | |  |  |  | |  | |  |  |  |  |  |  |  |
|  | Armstrong, BC | 50.41172, -119.19531 | 4 |  |  | |  | |  |  |  | |  | |  |  |  |  |  |  |  |
|  |  |  |  |  |  | |  | |  |  |  | |  | |  |  |  |  |  |  |  |
|  | Mara, BC | 50.65385, -119.09082 | 4 |  |  | |  | |  |  |  | |  | |  |  |  |  |  |  |  |
|  |  |  |  |  |  | |  | |  |  |  | |  | |  |  |  |  |  |  |  |
|  | Pemberton, BC | 50.42932, -122.90767 | 3 |  |  | |  | |  |  |  | |  | |  |  |  |  |  |  |  |
|  |  |  |  |  |  | |  | |  |  |  | |  | |  |  |  |  |  |  |  |
|  | Kelowna, BC | 49.82179, -119.44088 | 4 |  |  | |  | |  |  |  | |  | |  |  |  |  |  |  |  |
|  |  |  |  |  |  | |  | |  |  |  | |  | |  |  |  |  |  |  |  |
|  | Qualicum Beach, BC | 49.30970, -124.49929 | 5 |  |  | |  | |  |  |  | |  | |  |  |  |  |  |  |  |
|  |  |  |  |  |  | |  | |  |  |  | |  | |  |  |  |  |  |  |  |
|  | Coombs, BC | 49.30578, -124.40696 | 5 |  |  | |  | |  |  |  | |  | |  |  |  |  |  |  |  |
|  |  |  |  |  |  | |  | |  |  |  | |  | |  |  |  |  |  |  |  |
|  | Westholme, BC | 48.87465, -123.70599 | 4 |  |  | |  | |  |  |  | |  | |  |  |  |  |  |  |  |
|  |  |  |  |  |  | |  | |  |  |  | |  | |  |  |  |  |  |  |  |
|  | Duncan, BC | 48.73699, -123.66114 | 3 |  |  | |  | |  |  |  | |  | |  |  |  |  |  |  |  |
|  |  |  |  |  |  | |  | |  |  |  | |  | |  |  |  |  |  |  |  |
|  | Cobble Hill, BC | 48.71343, -123.59735 | 5 |  |  | |  | |  |  |  | |  | |  |  |  |  |  |  |  |
|  |  |  |  |  |  | |  | |  |  |  | |  | |  |  |  |  |  |  |  |
|  | Malahat Creek, BC | 48.61804, -123.52268 | 3 |  |  | |  | |  |  |  | |  | |  |  |  |  |  |  |  |
|  |  |  |  |  |  | |  | |  |  |  | |  | |  |  |  |  |  |  |  |
|  | Oliver, BC | 49.24608, -119.56255 | 1 |  |  | |  | |  |  |  | |  | |  |  |  |  |  |  |  |
|  |  |  |  |  |  | |  | |  |  |  | |  | |  |  |  |  |  |  |  |
|  | Salmo, BC | 49.18377, -117.2829 | 1 |  |  | |  | |  |  |  | |  | |  |  |  |  |  |  |  |
|  |  |  |  |  |  | |  | |  |  |  | |  | |  |  |  |  |  |  |  |
|  | Fort Steele, BC | 49.60934, -115.64057 | 3 |  |  | |  | |  |  |  | |  | |  |  |  |  |  |  |  |
|  |  |  |  |  |  | |  | |  |  |  | |  | |  |  |  |  |  |  |  |
|  | Johnston Creek West, BC | 49.04614, -119.06191 | 1 |  |  | |  | |  |  |  | |  | |  |  |  |  |  |  |  |
|  |  |  |  |  |  | |  | |  |  |  | |  | |  |  |  |  |  |  |  |
|  | Port Alberni, BC | 49.27277, -124.7679 | 3 |  |  | |  | |  |  |  | |  | |  |  |  |  |  |  |  |
|  |  |  |  |  |  | |  | |  |  |  | |  | |  |  |  |  |  |  |  |
|  | Kentville, Nova Scotia | 45.06297, -64.48433 | 3 |  |  | |  | |  |  |  | |  | |  |  |  |  |  |  |  |
|  |  |  |  |  |  | |  | |  |  |  | |  | |  |  |  |  |  |  |  |
|  | Balcombe South, Nova Scotia | 44.98169, -64.98908 | 3 | 3 |  | |  | |  |  |  | |  | |  |  |  |  |  |  |  |
|  |  |  |  |  |  | |  | |  |  |  | |  | |  |  |  |  |  |  |  |
|  | Killbride, Newfoundland | 47.54462, -52.89314 | 3 | 3 |  | |  | |  |  |  | |  | |  |  |  |  |  |  |  |
|  |  |  |  |  |  | |  | |  |  |  | |  | |  |  |  |  |  |  |  |
| *Agriotes obscurus* (Linnaeus) | Agassiz, BC | 49.24336, -121.7588 | 4 | 4 |  | | 1 | | 1 | 2 | 2 | | 2 | | 1 | 2 | 1 | 1 | 2 | 2 |  |
|  |  |  |  |  |  | |  | |  |  |  | |  | |  |  |  |  |  |  |  |
|  | Creston, BC | 49.09215, -116.46917 | 3 |  |  | |  | |  |  |  | |  | |  |  |  |  |  |  |  |
|  |  |  |  |  |  | |  | |  |  |  | |  | |  |  |  |  |  |  |  |
|  | Revelstoke, BC | 50.98448, -118.22681 | 3 |  |  | |  | |  |  |  | |  | |  |  |  |  |  |  |  |
|  |  |  |  |  |  | |  | |  |  |  | |  | |  |  |  |  |  |  |  |
|  | Salmon Arm, BC | 50.67931, -119.21733 | 3 |  |  | |  | |  |  |  | |  | |  |  |  |  |  |  |  |
|  |  |  |  |  |  | |  | |  |  |  | |  | |  |  |  |  |  |  |  |
|  | Pemberton, BC | 50.42932, -122.90767 | 3 |  |  | |  | |  |  |  | |  | |  |  |  |  |  |  |  |
|  |  |  |  |  |  | |  | |  |  |  | |  | |  |  |  |  |  |  |  |
|  | Rock Creek, BC | 49.12939, -118.9936 | 1 |  |  | |  | |  |  |  | |  | |  |  |  |  |  |  |  |
|  |  |  |  |  |  | |  | |  |  |  | |  | |  |  |  |  |  |  |  |
|  | Salmo, BC | 49.18378, -117.2829 | 1 |  |  | |  | |  |  |  | |  | |  |  |  |  |  |  |  |
|  |  |  |  |  |  | |  | |  |  |  | |  | |  |  |  |  |  |  |  |
|  | Enderby, BC | 50.55034, -119.1419 | 1 | 1 |  | |  | |  |  |  | |  | |  |  |  |  |  |  |  |
|  |  |  |  |  |  | |  | |  |  |  | |  | |  |  |  |  |  |  |  |
|  | Kentville, Nova Scotia | 45.06297, -64.48433 | 3 | 3 |  | |  | |  |  |  | | 1 | |  |  |  |  |  |  |  |
|  |  |  |  |  |  | |  | |  |  |  | |  | |  |  |  |  |  |  |  |
| *Agriotes sputator* (Linnaeus) | Cerom, Quebec | 45.57901, -73.24419 | 5 | 4 | 2 | | 1 | | 1 | 2 | 2 | | 1 | | 1 | 2 | 1 | 1 | 2 | 2 | 1 |
|  |  |  |  |  |  | |  | |  |  |  | |  | |  |  |  |  |  |  |  |
|  | Balcombe South, Nova Scotia | 44.98169, -64.98908 | 5 | 5 |  | |  | |  | 2 | 1 | | 1 | | 1 |  |  |  | 1 | 1 |  |
|  |  |  |  |  |  | |  | |  |  |  | |  | |  |  |  |  |  |  |  |
|  | Orwell Cove, Prince Edward Island | 46.13384, -62.86382 | 3 |  |  | |  | |  |  |  | |  | |  |  |  |  |  |  |  |
|  |  |  |  |  |  | |  | |  |  |  | |  | |  |  |  |  |  |  |  |
| *Agriotes mancus* (Say) | Cerom, Quebec | 45.57901, -73.24419 | 3 |  |  | |  | |  |  |  | |  | |  |  |  |  |  |  |  |
|  |  |  |  |  |  | |  | |  |  |  | |  | |  |  |  |  |  |  |  |
|  | Clarendon, Quebec | 45.62794, -76.51295 | 3 | 3 |  | |  | |  | 1 | 1 | | 1 | |  |  |  |  | 1 | 1 |  |
|  |  |  |  |  |  | |  | |  |  |  | |  | |  |  |  |  |  |  |  |
|  | Grand St. Esprit, Quebec | 46.15300, -72.45894 | 2 | 2 |  | |  | |  |  |  | |  | |  |  |  |  |  |  |  |
|  |  |  |  |  |  | |  | |  |  |  | |  | |  |  |  |  |  |  |  |
|  | Rodney, Ontario | 42.56220, -81.64782 | 3 | 3 | 2 | | 1 | | 1 | 2 | 2 | | 2 | | 1 | 1 |  | 1 | 2 | 2 | 1 |
|  |  |  |  |  |  | |  | |  |  |  | |  | |  |  |  |  |  |  |  |
|  | Ridgetown, Ontario | 42.44085, -81.89528 | 3 | 2 |  | |  | |  |  |  | |  | |  |  |  |  |  |  |  |
|  |  |  |  |  |  | |  | |  |  |  | |  | |  |  |  |  |  |  |  |
|  | Fredericton, New Brunswick | 45.92436, -66.60522 | 3 | 1 |  | |  | |  |  |  | |  | |  |  |  |  |  |  |  |
|  |  |  |  |  |  | |  | |  |  |  | |  | |  |  |  |  |  |  |  |
|  | Kentville, Nova Scotia | 45.06297, -64.48433 | 3 | 1 |  | |  | |  |  |  | |  | |  |  |  |  |  |  |  |
|  |  |  |  |  |  | |  | |  |  |  | |  | |  |  |  |  |  |  |  |
|  | Balcombe South, Nova Scotia | 44.98169, -64.98908 | 3 | 1 |  | |  | |  |  |  | |  | |  |  |  |  |  |  |  |
|  |  |  |  |  |  | |  | |  |  |  | |  | |  |  |  |  |  |  |  |
|  | Orwell Cove, Prince Edward Island | 46.13384, -62.86382 | 3 |  |  | |  | |  |  |  | |  | |  |  |  |  |  |  |  |
|  |  |  |  |  |  | |  | |  |  |  | |  | |  |  |  |  |  |  |  |
| *Agriotes pubescens* (Melsheimer) | Cerom, Quebec | 45.57901, -73.24419 | 3 |  |  | |  | |  |  |  | |  | |  |  |  |  |  |  |  |
|  |  |  |  |  |  | |  | |  |  |  | |  | |  |  |  |  |  |  |  |
|  | Clarendon, Quebec | 45.62794, -76.51295 | 3 | 3 |  | |  | |  |  |  | |  | |  |  |  |  |  |  |  |
|  |  |  |  |  |  | |  | |  |  |  | |  | |  |  |  |  |  |  |  |
|  | La visitation, Quebec | 46.08967, -72.63212 | 3 | 3 |  | |  | |  |  |  | |  | |  |  |  |  |  |  |  |
|  |  |  |  |  |  | |  | |  |  |  | |  | |  |  |  |  |  |  |  |
|  | Courtland, Ontario | 42.84054, -80.63238 | 3 |  |  | |  | |  |  |  | |  | |  |  |  |  |  |  |  |
|  |  |  |  |  |  | |  | |  |  |  | |  | |  |  |  |  |  |  |  |
|  | Jeannettes Creek, Ontario | 42.31982, -82.42138 | 4 | 4 | 1 | | 1 | | 1 | 1 | 2 | | 2 | | 1 | 3 | 1 | 1 | 3 | 3 | 1 |
|  |  |  |  |  |  | |  | |  |  |  | |  | |  |  |  |  |  |  |  |
|  | Ridegetown, Ontario | 42.44085, -81.89528 | 1 | 1 |  | |  | |  |  |  | |  | |  |  |  |  |  |  |  |
|  |  |  |  |  |  | |  | |  |  |  | |  | |  |  |  |  |  |  |  |
|  | Dresden, Ontatio | 42.58300, -82.18206 | 3 |  |  | |  | |  |  |  | |  | |  |  |  |  |  |  |  |
|  |  |  |  |  |  | |  | |  |  |  | |  | |  |  |  |  |  |  |  |
| *Agriotes ferrugineipennis* (*LeConte*) | Pemberton, BC | 50.42932, -122.90767 | 5 | 4 | 1 | | 1 | | 1 | 3 | 2 | | 3 | | 1 | 3 | 1 | 1 | 3 | 1 | 1 |
|  |  |  |  |  |  | |  | |  |  |  | |  | |  |  |  |  |  |  |  |
|  | Rock Creek, BC | 49.12936, -118.9936 | 3 |  |  | |  | |  |  |  | |  | |  |  |  |  |  |  |  |
|  |  |  |  |  |  | |  | |  |  |  | |  | |  |  |  |  |  |  |  |
|  | Salmon Arm, BC | 50.67931, -119.21733 | 3 |  |  | |  | |  |  |  | |  | |  |  |  |  |  |  |  |
|  |  |  |  |  |  | |  | |  |  |  | |  | |  |  |  |  |  |  |  |
|  | Grand Forks, BC | 49.01263, -118.49424 | 3 |  |  | |  | |  |  |  | |  | |  |  |  |  |  |  |  |
|  |  |  |  |  |  | |  | |  |  |  | |  | |  |  |  |  |  |  |  |
|  | Revelstoke, BC | 50.98448, -118.22681 | 3 |  |  | |  | |  |  |  | |  | |  |  |  |  |  |  |  |
|  |  |  |  |  |  | |  | |  |  |  | |  | |  |  |  |  |  |  |  |
|  | Kelowna, BC | 49.82179, -119.44088 | 3 |  |  | |  | |  |  |  | |  | |  |  |  |  |  |  |  |
|  |  |  |  |  |  | |  | |  |  |  | |  | |  |  |  |  |  |  |  |
|  | Oliver, BC | 49.24608, -119.56255 | 3 | 1 |  | |  | |  |  |  | |  | |  |  |  |  |  |  |  |
|  |  |  |  |  |  | |  | |  |  |  | |  | |  |  |  |  |  |  |  |
|  | Cawston, BC | 49.19055, -119.76856 | 3 |  |  | |  | |  |  |  | |  | |  |  |  |  |  |  |  |
|  |  |  |  |  |  | |  | |  |  |  | |  | |  |  |  |  |  |  |  |
| *Limonius agonus* (Say) | Ridgetown, Ontario | 42.44085, -81.89528 | 3 | 1 |  | |  | |  |  |  | |  | |  |  |  |  |  |  |  |
|  |  |  |  |  |  | |  | |  |  |  | |  | |  |  |  |  |  |  |  |
|  | Parkhill, Ontario | 43.16233, -81.6835 | 3 |  |  | |  | |  |  |  | |  | |  |  |  |  |  |  |  |
|  |  |  |  |  |  | |  | |  |  |  | |  | |  |  |  |  |  |  |  |
|  | Jeannettes Creek, Ontario | 42.31982, -82.42138 | 4 | 3 | 2 | |  | | 1 |  | 1 | | 1 | |  | 1 |  |  | 1 | 1 |  |
|  |  |  |  |  |  | |  | |  |  |  | |  | |  |  |  |  |  |  |  |
|  | La visitation, Quebec | 46.08967, -72.63212 | 3 | 1 |  | | 1 | |  | 2 | 1 | | 2 | | 1 | 2 |  | 1 | 2 | 2 |  |
|  |  |  |  |  |  | |  | |  |  |  | |  | |  |  |  |  |  |  |  |
| *Limonius californicus* (Mannerheim) | Oliver, BC | 49.24608, -119.56255 | 3 | 3 | 2 | |  | | 1 | 1 | 2 | |  | | 1 | 1 | 1 | 1 | 1 | 1 |  |
|  |  |  |  |  |  | |  | |  |  |  | |  | |  |  |  |  |  |  |  |
|  | Grand Forks, BC | 49.01263, -118.49425 | 3 |  |  | |  | |  |  |  | |  | |  |  |  |  |  |  |  |
|  |  |  |  |  |  | |  | |  |  |  | |  | |  |  |  |  |  |  |  |
|  | Fort Steele, BC | 49.60934, -115.64057 | 3 |  |  | |  | |  |  |  | |  | |  |  |  |  |  |  |  |
|  |  |  |  |  |  | |  | |  |  |  | |  | |  |  |  |  |  |  |  |
|  | Windermere, BC | 50.46635, -115.97995 | 3 |  |  | |  | |  |  |  | |  | |  |  |  |  |  |  |  |
|  |  |  |  |  |  | |  | |  |  |  | |  | |  |  |  |  |  |  |  |
|  | Johnston Creek West, BC | 49.04614, -119.06191 | 3 |  |  | |  | |  |  |  | |  | |  |  |  |  |  |  |  |
|  |  |  |  |  |  | |  | |  |  |  | |  | |  |  |  |  |  |  |  |
|  | Kelowna, BC | 49.82179, -119.44088 | 3 |  |  | |  | |  |  |  | |  | |  |  |  |  |  |  |  |
|  |  |  |  |  |  | |  | |  |  |  | |  | |  |  |  |  |  |  |  |
|  | Princeton, BC | 49.45570, -120.3552 | 3 |  |  | |  | |  |  |  | |  | |  |  |  |  |  |  |  |
|  |  |  |  |  |  | |  | |  |  |  | |  | |  |  |  |  |  |  |  |
|  | Rock Creek, BC | 49.12939, -118.9936 | 3 |  |  | |  | |  |  |  | |  | |  |  |  |  |  |  |  |
|  |  |  |  |  |  | |  | |  |  |  | |  | |  |  |  |  |  |  |  |
|  | Midway, BC | 49.01590, -118.80605 | 3 |  |  | |  | |  |  |  | |  | |  |  |  |  |  |  |  |
|  |  |  |  |  |  | |  | |  |  |  | |  | |  |  |  |  |  |  |  |
|  | Cawston, BC | 49.19055, -119.76856 | 3 |  |  | |  | |  |  |  | |  | |  |  |  |  |  |  |  |
|  |  |  |  |  |  | |  | |  |  |  | |  | |  |  |  |  |  |  |  |
|  | Claresholm, AB | 50.01910, -113.57992 | 3 | 3 |  | | 1 | |  | 1 | 1 | |  | |  | 2 |  |  | 2 | 2 |  |
|  |  |  |  |  |  | |  | |  |  |  | |  | |  |  |  |  |  |  |  |
|  | Olds, AB | 51.79477, -114.12071 | 3 | 1 |  | |  | |  |  |  | |  | |  |  |  |  |  |  |  |
|  |  |  |  |  |  | |  | |  |  |  | |  | |  |  |  |  |  |  |  |
|  | Manhattan, Montana | 45.86400, -111.32637 | 3 |  |  | |  | |  |  |  | |  | |  |  |  |  |  |  |  |
|  |  |  |  |  |  | |  | |  |  |  | |  | |  |  |  |  |  |  |  |
|  | Harrison Field, Montana | 45.70471, -111.78468 | 3 | 2 |  | |  | |  |  |  | |  | |  |  |  |  |  |  |  |
|  |  |  |  |  |  | |  | |  |  |  | |  | |  |  |  |  |  |  |  |
|  | Lenville, Idaho | 46.63888, -116.83865 | 3 |  |  | |  | |  |  |  | |  | |  |  |  |  |  |  |  |
|  |  |  |  |  |  | |  | |  |  |  | |  | |  |  |  |  |  |  |  |
|  | George, Washington | 47.07910, -119.8558 | 3 |  |  | |  | |  |  |  | |  | |  |  |  |  |  |  |  |
|  |  |  |  |  |  | |  | |  |  |  | |  | |  |  |  |  |  |  |  |
| *Limonius canus* (LeConte) | Oliver, BC | 49.24608, -119.56255 | 4 | 4 | 1 | | 1 | | 1 | 3 | 3 | | 2 | |  | 3 | 1 | 1 | 3 | 3 |  |
|  |  |  |  |  |  | |  | |  |  |  | |  | |  |  |  |  |  |  |  |
|  | Pemberton, BC | 50.42932, -122.90767 | 3 |  |  | |  | |  |  |  | |  | |  |  |  |  |  |  |  |
|  |  |  |  |  |  | |  | |  |  |  | |  | |  |  |  |  |  |  |  |
|  | Cawston, BC | 49.19055, -119.76856 | 3 |  |  | |  | |  |  |  | |  | |  |  |  |  |  |  |  |
|  |  |  |  |  |  | |  | |  |  |  | |  | |  |  |  |  |  |  |  |
|  | Revelstoke, BC | 50.98448, -118.22681 | 4 | 2 |  | |  | |  |  |  | |  | |  |  |  |  |  |  |  |
|  |  |  |  |  |  | |  | |  |  |  | |  | |  |  |  |  |  |  |  |
|  | Kelowna, BC | 49.82179, -119.44088 | 3 |  |  | |  | |  |  |  | |  | |  |  |  |  |  |  |  |
|  |  |  |  |  |  | |  | |  |  |  | |  | |  |  |  |  |  |  |  |
|  | George, Washington | 47.07910, -119.8558 | 3 |  |  | |  | |  |  |  | |  | |  |  |  |  |  |  |  |
|  |  |  |  |  |  | |  | |  |  |  | |  | |  |  |  |  |  |  |  |
|  | Pasco, Washington | 46.23024, -119.09232 | 3 |  |  | |  | |  |  |  | |  | |  |  |  |  |  |  |  |
|  |  |  |  |  |  | |  | |  |  |  | |  | |  |  |  |  |  |  |  |
|  | Hermiston, Oregon | 45.84023, -119.28956 | 3 |  |  | |  | |  |  |  | |  | |  |  |  |  |  |  |  |
|  |  |  |  |  |  | |  | |  |  |  | |  | |  |  |  |  |  |  |  |
|  | Applegate, California | 39.00152, -120.99134 | 3 |  |  | |  | |  |  |  | |  | |  |  |  |  |  |  |  |
|  |  |  |  |  |  | |  | |  |  |  | |  | |  |  |  |  |  |  |  |
| *Limonius infuscatus* (Motschulsky) | Oliver, BC | 49.24608, -119.56255 | 4 | 3 | 2 | | 1 | | 1 | 4 | 3 | | 3 | | 1 | 3 | 1 | 1 |  | 2 | 1 |
|  |  |  |  |  |  | |  | |  |  |  | |  | |  |  |  |  |  |  |  |
|  | Johnston Creek West, BC | 49.04614, -119.06191 | 5 |  |  | |  | |  |  |  | |  | |  |  |  |  |  |  |  |
|  |  |  |  |  |  | |  | |  |  |  | |  | |  |  |  |  |  |  |  |
|  | Grand Forks, BC | 49.01263, -118.49425 | 5 |  |  | |  | |  |  |  | |  | |  |  |  |  |  |  |  |
|  |  |  |  |  |  | |  | |  |  |  | |  | |  |  |  |  |  |  |  |
|  | Salmo, BC | 49.18378, -117.2829 | 4 |  |  | |  | |  |  |  | |  | |  |  |  |  |  |  |  |
|  |  |  |  |  |  | |  | |  |  |  | |  | |  |  |  |  |  |  |  |
|  | Creston, BC | 49.09215, -116.46917 | 5 |  |  | |  | |  |  |  | |  | |  |  |  |  |  |  |  |
|  |  |  |  |  |  | |  | |  |  |  | |  | |  |  |  |  |  |  |  |
|  | Revelstoke, BC | 50.98448, -118.22681 | 4 |  |  | |  | |  |  |  | |  | |  |  |  |  |  |  |  |
|  |  |  |  |  |  | |  | |  |  |  | |  | |  |  |  |  |  |  |  |
|  | Salmon Arm, BC | 50.67931, -119.21733 | 5 |  |  | |  | |  |  |  | |  | |  |  |  |  |  |  |  |
|  |  |  |  |  |  | |  | |  |  |  | |  | |  |  |  |  |  |  |  |
|  | Armstrong, BC | 50.41172, -119.19531 | 5 |  |  | |  | |  |  |  | |  | |  |  |  |  |  |  |  |
|  |  |  |  |  |  | |  | |  |  |  | |  | |  |  |  |  |  |  |  |
|  | Midway, BC | 49.01590, -118.80605 | 4 |  |  | |  | |  |  |  | |  | |  |  |  |  |  |  |  |
|  |  |  |  |  |  | |  | |  |  |  | |  | |  |  |  |  |  |  |  |
|  | Malakwa, BC | 50.95443, -118.76123 | 3 |  |  | |  | |  |  |  | |  | |  |  |  |  |  |  |  |
|  |  |  |  |  |  | |  | |  |  |  | |  | |  |  |  |  |  |  |  |
|  | Princeton, BC | 49.45570, -120.3552 | 3 |  |  | |  | |  |  |  | |  | |  |  |  |  |  |  |  |
|  |  |  |  |  |  | |  | |  |  |  | |  | |  |  |  |  |  |  |  |
|  | Cawston, BC | 49.19055, -119.76856 | 1 |  |  | |  | |  |  |  | |  | |  |  |  |  |  |  |  |
|  |  |  |  |  |  | |  | |  |  |  | |  | |  |  |  |  |  |  |  |
|  | Kelowna, BC | 49.82179, -119.44088 | 4 |  |  | |  | |  |  |  | |  | |  |  |  |  |  |  |  |
|  |  |  |  |  |  | |  | |  |  |  | |  | |  |  |  |  |  |  |  |
|  | Cobble Hill, BC | 48.71343, -123.59735 | 3 |  |  | |  | |  |  |  | |  | |  |  |  |  |  |  |  |
|  |  |  |  |  |  | |  | |  |  |  | |  | |  |  |  |  |  |  |  |
|  | Foremost, Alberta | 49.474732, -111.44894 | 1 | 1 |  | |  | |  |  |  | |  | |  |  |  |  |  |  |  |
|  |  |  |  |  |  | |  | |  |  |  | |  | |  |  |  |  |  |  |  |
|  | Manhattan, Montana | 45.86400, -111.32637 | 3 |  |  | |  | |  |  |  | |  | |  |  |  |  |  |  |  |
|  |  |  |  |  |  | |  | |  |  |  | |  | |  |  |  |  |  |  |  |
|  | Moscow, Idaho | 46.72187, -117.01737 | 3 |  |  | |  | |  |  |  | |  | |  |  |  |  |  |  |  |
|  |  |  |  |  |  | |  | |  |  |  | |  | |  |  |  |  |  |  |  |
|  | Sandpoint, Idaho | 48.27697, -116.55512 | 3 |  |  | |  | |  |  |  | |  | |  |  |  |  |  |  |  |
|  |  |  |  |  |  | |  | |  |  |  | |  | |  |  |  |  |  |  |  |
| *Selatosomus aeripennis destructor* (Brown) | Rose Prairie, BC | 56.52229, -120.82562 | 4 | 2 |  | |  | |  |  |  | |  | |  |  |  |  |  |  |  |
|  |  |  |  |  |  | |  | |  |  |  | |  | |  |  |  |  |  |  |  |
|  | Fort Vermillion, BC | 58.38771, -116.01745 | 4 | 4 |  | |  | |  |  |  | |  | |  |  |  |  |  |  |  |
|  |  |  |  |  |  | |  | |  |  |  | |  | |  |  |  |  |  |  |  |
|  | Fourth Creek, Alberta | 56.07233, -118.93314 | 4 | 4 |  | |  | |  |  |  | |  | |  |  |  |  |  |  |  |
|  |  |  |  |  |  | |  | |  |  |  | |  | |  |  |  |  |  |  |  |
|  | Buffalo Head Prairie, Alberta | 58.06621, -116.34948 | 4 | 4 |  | |  | |  |  |  | |  | |  |  |  |  |  |  |  |
|  |  |  |  |  |  | |  | |  |  |  | |  | |  |  |  |  |  |  |  |
|  | Trochu, Alberta | 51.82404, -113.22681 | 3 | 3 |  | |  | |  |  |  | |  | |  |  |  |  |  |  |  |
|  |  |  |  |  |  | |  | |  |  |  | |  | |  |  |  |  |  |  |  |
|  | Medicine Hat, Alberta | 50.02573, -110.68197 | 3 | 2 |  | |  | |  |  |  | |  | |  |  |  |  |  |  |  |
|  |  |  |  |  |  | |  | |  |  |  | |  | |  |  |  |  |  |  |  |
|  | Jean Côté, Alberta | 55.89858, -117.31027 | 3 | 2 |  | |  | |  |  |  | |  | |  |  |  |  |  |  |  |
|  |  |  |  |  |  | |  | |  |  |  | |  | |  |  |  |  |  |  |  |
|  | Vulcan, Alberta | 50.40321, -113.27019 | 3 | 3 | 2 | | 1 | | 1 | 2 | 3 | |  | | 1 | 3 |  | 1 | 2 | 2 | 1 |
|  |  |  |  |  |  | |  | |  |  |  | |  | |  |  |  |  |  |  |  |
|  | Colonsay, Saskatchewan | 51.97713, -105.87542 | 3 | 2 |  | |  | |  |  |  | |  | |  |  |  |  |  |  |  |
|  |  |  |  |  |  | |  | |  |  |  | |  | |  |  |  |  |  |  |  |
|  | Eston, Saskatchewan | 51.15053, -108.75789 | 3 | 3 |  | |  | |  |  |  | |  | |  |  |  |  |  |  |  |
|  |  |  |  |  |  | |  | |  |  |  | |  | |  |  |  |  |  |  |  |
|  | Swift Current, Saskatchewan | 50.28707, -107.7558 | 3 | 3 |  | |  | |  |  |  | |  | |  |  |  |  |  |  |  |
|  |  |  |  |  |  | |  | |  |  |  | |  | |  |  |  |  |  |  |  |
|  | Blaine Lake, Saskatchewan | 52.82832, -106.88062 | 1 | 1 |  | |  | |  |  |  | |  | |  |  |  |  |  |  |  |
|  |  |  |  |  |  | |  | |  |  |  | |  | |  |  |  |  |  |  |  |
|  | Bluesky, Alberta | 56.07463, -118.24064 | 4 | 3 |  | |  | |  |  |  | |  | |  |  |  |  |  |  |  |
|  |  |  |  |  |  | |  | |  |  |  | |  | |  |  |  |  |  |  |  |
|  | Congress, Saskatchewan | 49.75694, -106.02585 | 1 | 1 |  | |  | |  |  |  | |  | |  |  |  |  |  |  |  |
|  |  |  |  |  |  | |  | |  |  |  | |  | |  |  |  |  |  |  |  |
|  | Fairview, Alberta | 55.96267, -118.71072 | 4 | 4 |  | |  | |  |  |  | |  | |  |  |  |  |  |  |  |
|  |  |  |  |  |  | |  | |  |  |  | |  | |  |  |  |  |  |  |  |
|  | Foremost, Alberta | 49.47473, -111.44894 | 3 | 3 |  | |  | |  |  |  | |  | |  |  |  |  |  |  |  |
|  |  |  |  |  |  | |  | |  |  |  | |  | |  |  |  |  |  |  |  |
|  | Glidden, Saskatchewan | 51.23706, -109.16675 | 3 | 3 |  | |  | |  |  |  | |  | |  |  |  |  |  |  |  |
|  |  |  |  |  |  | |  | |  |  |  | |  | |  |  |  |  |  |  |  |
|  | Gull Lake, Saskatchewan | 50.09685, -108.48546 | 3 | 2 |  | |  | |  |  |  | |  | |  |  |  |  |  |  |  |
|  |  |  |  |  |  | |  | |  |  |  | |  | |  |  |  |  |  |  |  |
|  | Lucky Lake, Saskatchewan | 50.98532, -107.13327 | 3 | 3 |  | |  | |  |  |  | |  | |  |  |  |  |  |  |  |
|  |  |  |  |  |  | |  | |  |  |  | |  | |  |  |  |  |  |  |  |
|  | Wrentham, Alberta | 49.52348, -112.17219 | 3 | 3 |  | |  | |  |  |  | |  | |  |  |  |  |  |  |  |
|  |  |  |  |  |  | |  | |  |  |  | |  | |  |  |  |  |  |  |  |
| *Hypnoidus bicolor* (Eschscholtz) | Rose Prairie, BC | 56.52229, -120.82562 | 6 | 6 |  | |  | |  |  |  | |  | |  |  |  |  |  |  |  |
|  |  |  |  |  |  | |  | |  |  |  | |  | |  |  |  |  |  |  |  |
|  | Buffalo Head Prairie, Alberta | 58.06621, -116.34948 | 4 | 4 |  | |  | |  |  |  | |  | |  |  |  |  |  |  |  |
|  |  |  |  |  |  | |  | |  |  |  | |  | |  |  |  |  |  |  |  |
|  | Standoff, Alberta | 49.46842, -113.30547 | 3 | 1 |  | |  | |  |  |  | |  | |  |  |  |  |  |  |  |
|  |  |  |  |  |  | |  | |  |  |  | |  | |  |  |  |  |  |  |  |
|  | Cluny, Alberta | 50.83665, -112.86691 | 3 | 1 |  | |  | |  |  |  | |  | |  |  |  |  |  |  |  |
|  |  |  |  |  |  | |  | |  |  |  | |  | |  |  |  |  |  |  |  |
|  | Drumhellar, Alberta | 51.46175, -112.70658 | 4 | 3 |  | |  | |  |  |  | |  | |  |  |  |  |  |  |  |
|  |  |  |  |  |  | |  | |  |  |  | |  | |  |  |  |  |  |  |  |
|  | Brooks, Alberta | 50.56565, -111.89758 | 3 | 3 |  | |  | |  |  |  | |  | |  |  |  |  |  |  |  |
|  |  |  |  |  |  | |  | |  |  |  | |  | |  |  |  |  |  |  |  |
|  | Cowley, Alberta | 49.56759, -114.0742 | 3 | 2 |  | |  | |  |  |  | |  | |  |  |  |  |  |  |  |
|  |  |  |  |  |  | |  | |  |  |  | |  | |  |  |  |  |  |  |  |
|  | Hines Creek, Alberta | 56.24712, -118.60497 | 4 | 4 |  | |  | |  |  |  | |  | |  |  |  |  |  |  |  |
|  |  |  |  |  |  | |  | |  |  |  | |  | |  |  |  |  |  |  |  |
|  | North Battleford, Saskatchewan | 52.77663, -108.30003 | 3 | 3 |  | |  | |  |  |  | |  | |  |  |  |  |  |  |  |
|  |  |  |  |  |  | |  | |  |  |  | |  | |  |  |  |  |  |  |  |
|  | Qu’Appelle, Saskatchewan | 50.54255, -103.87701 | 3 | 3 |  | |  | |  |  |  | |  | |  |  |  |  |  |  |  |
|  |  |  |  |  |  | |  | |  |  |  | |  | |  |  |  |  |  |  |  |
|  | Waskada, Manitoba | 49.09762, -100.80162 | 3 | 2 |  | |  | |  |  |  | |  | |  |  |  |  |  |  |  |
|  |  |  |  |  |  | |  | |  |  |  | |  | |  |  |  |  |  |  |  |
|  | Teulon, Manitoba | 50.38904, -97.26115 | 3 | 3 |  | |  | |  |  |  | |  | |  |  |  |  |  |  |  |
|  |  |  |  |  |  | |  | |  |  |  | |  | |  |  |  |  |  |  |  |
|  | Orangeville, Ontario | 43.93161, -80.10663 | 3 | 3 |  | |  | |  |  |  | |  | |  |  |  |  |  |  |  |
|  |  |  |  |  |  | |  | |  |  |  | |  | |  |  |  |  |  |  |  |
|  | Brookdale, Manitoba | 50.01806, -99.50086 | 3 | 2 |  | |  | |  |  |  | |  | |  |  |  |  |  |  |  |
|  |  |  |  |  |  | |  | |  |  |  | |  | |  |  |  |  |  |  |  |
|  | Congress, Saskatchewan | 49.75694, -106.02585 | 1 | 1 |  | |  | |  |  |  | |  | |  |  |  |  |  |  |  |
|  |  |  |  |  |  | |  | |  |  |  | |  | |  |  |  |  |  |  |  |
|  | Courtland, Ontario | 42.84054, -80.63238 | 3 |  |  | |  | |  |  |  | |  | |  |  |  |  |  |  |  |
|  |  |  |  |  |  | |  | |  |  |  | |  | |  |  |  |  |  |  |  |
|  | Eston, Saskatchewan | 51.14770, -108.75682 | 3 | 3 |  | |  | |  |  |  | |  | |  |  |  |  |  |  |  |
|  |  |  |  |  |  | |  | |  |  |  | |  | |  |  |  |  |  |  |  |
|  | Flatrock, British Columbia | 56.25852, -120.42973 | 4 | 4 |  | |  | |  |  |  | |  | |  |  |  |  |  |  |  |
|  |  |  |  |  |  | |  | |  |  |  | |  | |  |  |  |  |  |  |  |
|  | Hawarden, Saskatchewan | 51.41343, -106.6016 | 3 | 3 |  | |  | |  |  |  | |  | |  |  |  |  |  |  |  |
|  |  |  |  |  |  | |  | |  |  |  | |  | |  |  |  |  |  |  |  |
|  | Huxley, Alberta | 51.92133, -113.23332 | 3 | 3 |  | |  | |  |  |  | |  | |  |  |  |  |  |  |  |
|  |  |  |  |  |  | |  | |  |  |  | |  | |  |  |  |  |  |  |  |
|  | Jean Cote, Alberta | 55.89858, -117.31027 | 3 | 3 |  | |  | |  |  |  | |  | |  |  |  |  |  |  |  |
|  |  |  |  |  |  | |  | |  |  |  | |  | |  |  |  |  |  |  |  |
|  | Magrath, Alberta | 49.41573, -112.86385 | 2 | 2 |  | |  | |  |  |  | |  | |  |  |  |  |  |  |  |
|  |  |  |  |  |  | |  | |  |  |  | |  | |  |  |  |  |  |  |  |
|  | Medicine Hat, Alberta | 50.05162, -110.70923 | 6 | 5 |  | |  | |  |  |  | |  | |  |  |  |  |  |  |  |
|  |  |  |  |  |  | |  | |  |  |  | |  | |  |  |  |  |  |  |  |
|  | Rolling Hills, Alberta | 50.22269, -111.76866 | 4 | 4 |  | |  | |  |  |  | |  | |  |  |  |  |  |  |  |
|  |  |  |  |  |  | |  | |  |  |  | |  | |  |  |  |  |  |  |  |
|  | Rosetown, Saskatchewan | 51.55458, -107.99005 | 3 | 3 |  | |  | |  |  |  | |  | |  |  |  |  |  |  |  |
|  |  |  |  |  |  | |  | |  |  |  | |  | |  |  |  |  |  |  |  |
|  | Russell, Manitoba | 50.77997, -101.28241 | 3 | 3 |  | |  | |  |  |  | |  | |  |  |  |  |  |  |  |
|  |  |  |  |  |  | |  | |  |  |  | |  | |  |  |  |  |  |  |  |
|  | Torrington, Alberta | 51.79453, -113.60427 | 3 | 3 |  | |  | |  |  |  | |  | |  |  |  |  |  |  |  |
|  |  |  |  |  |  | |  | |  |  |  | |  | |  |  |  |  |  |  |  |
|  | Trochu, Alberta | 51.82847, -113.22926 | 3 | 2 |  | |  | |  |  |  | |  | |  |  |  |  |  |  |  |
|  |  |  |  |  |  | |  | |  |  |  | |  | |  |  |  |  |  |  |  |
|  | Valhalla, Alberta | 55.49164, -119.34855 | 4 | 3 |  | |  | |  |  |  | |  | |  |  |  |  |  |  |  |
|  |  |  |  |  |  | |  | |  |  |  | |  | |  |  |  |  |  |  |  |
|  | Whitemud, Alberta | 55.45330, -117.23435 | 4 | 2 |  | |  | |  |  |  | |  | |  |  |  |  |  |  |  |
|  |  |  |  |  |  | |  | |  |  |  | |  | |  |  |  |  |  |  |  |
|  | Winkler, Manitoba | 49.19575, -97.93922 | 3 | 3 |  | |  | |  |  |  | |  | |  |  |  |  |  |  |  |
|  |  |  |  |  |  | |  | |  |  |  | |  | |  |  |  |  |  |  |  |
| *Hypnoidus abbreviatus* (Say) | Courtland, Ontario | 42.84054, -80.63238 | 3 | 3 |  | |  | |  |  |  | |  | |  |  |  |  |  |  |  |
|  |  |  |  |  |  | |  | |  |  |  | |  | |  |  |  |  |  |  |  |
|  | Ridgetown, Ontario | 42.44085, -81.89528 | 7 | 6 |  | |  | |  |  |  | |  | |  |  |  |  |  |  |  |
|  |  |  |  |  |  | |  | |  |  |  | |  | |  |  |  |  |  |  |  |
|  | Brigham, Quebec | 45.24701, -72.84437 | 4 | 3 |  | |  | |  |  |  | |  | |  |  |  |  |  |  |  |
|  |  |  |  |  |  | |  | |  |  |  | |  | |  |  |  |  |  |  |  |
|  | Fredericton, New Brunswick | 45.92436, -66.60522 | 3 | 3 |  | |  | |  |  |  | |  | |  |  |  |  |  |  |  |
|  |  |  |  |  |  | |  | |  |  |  | |  | |  |  |  |  |  |  |  |
|  | Orwell Cove, Prince Edward Island | 46.13384,-62.86382 | 3 | 1 |  | |  | |  |  |  | |  | |  |  |  |  |  |  |  |
|  |  |  |  |  |  | |  | |  |  |  | |  | |  |  |  |  |  |  |  |
|  | Sainte Marie Salome , Quebec | 45.93163, -73.49725 | 3 | 3 |  | |  | |  |  |  | |  | |  |  |  |  |  |  |  |
|  |  |  |  |  |  | |  | |  |  |  | |  | |  |  |  |  |  |  |  |
| *Hadromorphus glaucus* (Germar) | Del Bonita, Alberta | 49.02879, -112.78551 | 5 | 5 |  | |  | |  |  |  | |  | |  |  |  |  |  |  |  |
|  |  |  |  |  |  | |  | |  |  |  | |  | |  |  |  |  |  |  |  |
| *Athous haemorrhoidalis* (Fabricius) | Jericho Beach, BC | 49.27208, -123.19448 | 3 | 2 |  | |  | |  |  |  | |  | |  |  |  |  |  |  |  |
|  |  |  |  |  |  | |  | |  |  |  | |  | |  |  |  |  |  |  |  |
|  | Ottawa, BC | 45.42151, -75.69775 | 3 | 2 |  | |  | |  |  |  | |  | |  |  |  |  |  |  |  |
|  |  |  |  |  |  | |  | |  |  |  | |  | |  |  |  |  |  |  |  |
| **Non-Target Species** |  |  |  |  |  | |  | |  |  |  | |  | |  |  |  |  |  |  |  |
| **Elateridae** |  |  |  |  |  | |  | |  |  |  | |  | |  |  |  |  |  |  |  |
| *Agriotes stabilis* (LeConte) | Boissevain, Manitoba | 49.23044, -100.05881 | 3 | 3 |  | |  | |  |  |  | |  | |  |  |  |  |  |  |  |
|  |  |  |  |  |  | |  | |  |  |  | |  | |  |  |  |  |  |  |  |
| *Agriotes sparsus* (LeConte) | Delta, BC | 49.10331, -123.0222 | 4 |  |  | |  | |  |  |  | |  | |  |  |  |  |  |  |  |
|  |  |  |  |  |  | |  | |  |  |  | |  | |  |  |  |  |  |  |  |
| *Agriotes oregonensis* Becker | Creston, BC | 49.09215, -116.46917 | 4 |  |  | |  | |  |  |  | |  | |  |  |  |  |  |  |  |
|  |  |  |  |  |  | |  | |  |  |  | |  | |  |  |  |  |  |  |  |
|  | Armstrong, BC | 50.41172, -119.19531 | 4 |  |  | |  | |  |  |  | |  | |  |  |  |  |  |  |  |
|  |  |  |  |  |  | |  | |  |  |  | |  | |  |  |  |  |  |  |  |
|  | Enderby, BC | 50.55034, -119.1419 | 3 | 1 |  | |  | |  |  |  | |  | |  |  |  |  |  |  |  |
|  |  |  |  |  |  | |  | |  |  |  | |  | |  |  |  |  |  |  |  |
| *Agriotes opaculus* (LeConte) | Princeton, BC | 49.45570, -120.3552 | 3 |  |  | |  | |  |  |  | |  | |  |  |  |  |  |  |  |
|  |  |  |  |  |  | |  | |  |  |  | |  | |  |  |  |  |  |  |  |
| *Agriotes criddlei* (Van Dyke) | Princeton, BC | 49.45570, -120.3552 | 4 |  |  | |  | |  |  |  | |  | |  |  |  |  |  |  |  |
|  |  |  |  |  |  | |  | |  |  |  | |  | |  |  |  |  |  |  |  |
| *Dalopius maritimus* Brown | Pemberton, BC | 50.42932, -122.90767 | 4 |  |  | |  | |  |  |  | |  | |  |  |  |  |  |  |  |
|  |  |  |  |  |  | |  | |  |  |  | |  | |  |  |  |  |  |  |  |
|  | Delta, BC | 49.10331, -123.0222 | 3 | 3 |  | |  | |  |  |  | |  | |  |  |  |  |  |  |  |
|  |  |  |  |  |  | |  | |  |  |  | |  | |  |  |  |  |  |  |  |
| *Dalopius corvinus* Brown | Rock Creek, BC | 49.12939, -118.9936 | 3 |  |  | |  | |  |  |  | |  | |  |  |  |  |  |  |  |
|  |  |  |  |  |  | |  | |  |  |  | |  | |  |  |  |  |  |  |  |
| *Dalopius asellus* Brown | Guy, Alberta | 55.54948, -117.12967 | 4 |  |  | |  | |  |  |  | |  | |  |  |  |  |  |  |  |
|  |  |  |  |  |  | |  | |  |  |  | |  | |  |  |  |  |  |  |  |
|  | Beaverlodge, Alberta | 55.21001, -119.42994 | 2 | 2 |  | |  | |  |  |  | |  | |  |  |  |  |  |  |  |
|  |  |  |  |  |  | |  | |  |  |  | |  | |  |  |  |  |  |  |  |
| *Dalopius pallidus* Brown | Orangeville, Ontario | 43.93161, -80.10663 | 2 |  |  | |  | |  |  |  | |  | |  |  |  |  |  |  |  |
|  |  |  |  |  |  | |  | |  |  |  | |  | |  |  |  |  |  |  |  |
|  | Ponoka, Alberta | 52.67602, -113.58332 | 3 |  |  | |  | |  |  |  | |  | |  |  |  |  |  |  |  |
|  |  |  |  |  |  | |  | |  |  |  | |  | |  |  |  |  |  |  |  |
|  | Benito, Manitoba | 51.90969, -101.53843 | 3 |  |  | |  | |  |  |  | |  | |  |  |  |  |  |  |  |
|  |  |  |  |  |  | |  | |  |  |  | |  | |  |  |  |  |  |  |  |
|  | Kamarousk, Quebec | 47.56815, -69.86463 | 3 |  |  | |  | |  |  |  | |  | |  |  |  |  |  |  |  |
|  |  |  |  |  |  | |  | |  |  |  | |  | |  |  |  |  |  |  |  |
| *Gambrinus ursinus* (Van Dyke) | Claresholm, Alberta | 50.01910, -113.57992 | 3 |  |  | |  | |  |  |  | |  | |  |  |  |  |  |  |  |
|  |  |  |  |  |  | |  | |  |  |  | |  | |  |  |  |  |  |  |  |
| *Corymbitodes lobatus* (Eschscholtz) | Delta, BC | 49.10331, -123.0222 | 3 | 1 |  | |  | |  |  |  | |  | |  |  |  |  |  |  |  |
|  |  |  |  |  |  | |  | |  |  |  | |  | |  |  |  |  |  |  |  |
|  | Pemberton, BC | 50.42932, -122.90767 | 4 | 3 |  | |  | |  |  |  | |  | |  |  |  |  |  |  |  |
|  |  |  |  |  |  | |  | |  |  |  | |  | |  |  |  |  |  |  |  |
| *Selatosomus festivus* (LeConte) | Burnaby Lake, BC | 49.24479, -122.93077 | 1 |  |  | |  | |  |  |  | |  | |  |  |  |  |  |  |  |
|  |  |  |  |  |  | |  | |  |  |  | |  | |  |  |  |  |  |  |  |
|  | Pacific Spirit Park, BC | 49.27289, -123.22972 | 3 |  |  | |  | |  |  |  | |  | |  |  |  |  |  |  |  |
|  |  |  |  |  |  | |  | |  |  |  | |  | |  |  |  |  |  |  |  |
| *Selatosomus suckleyi* (LeConte) | Aldergrove Regional Park, BC | 49.01164, -122.46563 | 2 |  |  | |  | |  |  |  | |  | |  |  |  |  |  |  |  |
|  |  |  |  |  |  | |  | |  |  |  | |  | |  |  |  |  |  |  |  |
| *Prosternon bombycinum* (Germar) | Creston, BC | 49.09215, -116.46917 | 3 |  |  | |  | |  |  |  | |  | |  |  |  |  |  |  |  |
|  |  |  |  |  |  | |  | |  |  |  | |  | |  |  |  |  |  |  |  |
| *Tesolasomus morulus* (LeConte) | Brigade Lake, BC | 49.26989, -125.28102 | 2 |  |  | |  | |  |  |  | |  | |  |  |  |  |  |  |  |
|  |  |  |  |  |  | |  | |  |  |  | |  | |  |  |  |  |  |  |  |
| *Stropenron pudica* (Brown) | Salmon Arm, BC | 50.67931, -119.21733 | 1 |  |  | |  | |  |  |  | |  | |  |  |  |  |  |  |  |
|  |  |  |  |  |  | |  | |  |  |  | |  | |  |  |  |  |  |  |  |
|  | Whytecliff, BC | 49.37432, -123.28927 | 1 |  |  | |  | |  |  |  | |  | |  |  |  |  |  |  |  |
|  |  |  |  |  |  | |  | |  |  |  | |  | |  |  |  |  |  |  |  |
|  | Soda Creek, BC | 52.35066, -122.28321 | 1 |  |  | |  | |  |  |  | |  | |  |  |  |  |  |  |  |
|  |  |  |  |  |  | |  | |  |  |  | |  | |  |  |  |  |  |  |  |
| *Ampedus behrensi* (Horn) | Pacific Spirit Park, BC | 49.27289, -123.22972 | 3 |  |  | |  | |  |  |  | |  | |  |  |  |  |  |  |  |
|  |  |  |  |  |  | |  | |  |  |  | |  | |  |  |  |  |  |  |  |
|  | Burnaby Lake, BC | 49.24479, -122.92077 | 3 |  |  | |  | |  |  |  | |  | |  |  |  |  |  |  |  |
|  |  |  |  |  |  | |  | |  |  |  | |  | |  |  |  |  |  |  |  |
| *Ampedus rhodopus* (LeConte) | Malcolm Knapp Research Forest, BC | 49.26367, -122.57319 | 3 |  |  | |  | |  |  |  | |  | |  |  |  |  |  |  |  |
|  |  |  |  |  |  | |  | |  |  |  | |  | |  |  |  |  |  |  |  |
| *Melanotus communis* (Gyllenhal) | Ridgetown, Ontario | 42.44085, -81.89528 | 7 | 6 |  | |  | |  |  |  | |  | |  |  |  |  |  |  |  |
|  |  |  |  |  |  | |  | |  |  |  | |  | |  |  |  |  |  |  |  |
| *Melanotus depressus* (Melsheimer) | Ridgetown, Ontario | 42.44085, -81.89528 | 6 | 6 |  | |  | |  |  |  | |  | |  |  |  |  |  |  |  |
|  |  |  |  |  |  | |  | |  |  |  | |  | |  |  |  |  |  |  |  |
| *Melanotus similis* (Kirby) | Ridgetown, Ontario | 42.44085, -81.89528 | 6 | 5 |  | |  | |  |  |  | |  | |  |  |  |  |  |  |  |
|  |  |  |  |  |  | |  | |  |  |  | |  | |  |  |  |  |  |  |  |
|  | Saint-Ambroise, Quebec | 48.55533, -71.32444 | 2 | 2 |  | |  | |  |  |  | |  | |  |  |  |  |  |  |  |
|  |  |  |  |  |  | |  | |  |  |  | |  | |  |  |  |  |  |  |  |
|  | Saint-Hyacinthe, Quebec | 45.63379, -72.9528 | 1 | 1 |  | |  | |  |  |  | |  | |  |  |  |  |  |  |  |
|  |  |  |  |  |  | |  | |  |  |  | |  | |  |  |  |  |  |  |  |
| *Melanotus morosus* (Candèze) | Ridgetown, Ontario | 42.44085, -81.89528 | 1 | 1 |  | |  | |  |  |  | |  | |  |  |  |  |  |  |  |
|  |  |  |  |  |  | |  | |  |  |  | |  | |  |  |  |  |  |  |  |
| *Hemicrepidius bilobatus* (Say) | Ridgetown, Ontario | 42.44085, -81.89528 | 2 |  |  | |  | |  |  |  | |  | |  |  |  |  |  |  |  |
|  |  |  |  |  |  | |  | |  |  |  | |  | |  |  |  |  |  |  |  |
| *Hemicrepidius memnonius* (Herbst) | Ridgetown, Ontario | 42.44085, 081.89528 | 1 |  |  | |  | |  |  |  | |  | |  |  |  |  |  |  |  |
|  |  |  |  |  |  | |  | |  |  |  | |  | |  |  |  |  |  |  |  |
| *Sylvanelater cylindriformis* (Herbst) | Orwell Cove, Prince Edward Island | 46.13384, -62.86382 | 3 |  |  | |  | |  |  |  | |  | |  |  |  |  |  |  |  |
|  |  |  |  |  |  | |  | |  |  |  | |  | |  |  |  |  |  |  |  |
| **Carabidae** |  |  |  |  |  | |  | |  |  |  | |  | |  |  |  |  |  |  |  |
| *Carabus granulatus* (Linnaeus) | Agassiz, BC | 49.24336, -121.7588 | 2 |  |  | |  | |  |  |  | |  | |  |  |  |  |  |  |  |
|  |  |  |  |  |  | |  | |  |  |  | |  | |  |  |  |  |  |  |  |
|  | Revelstoke, BC | 50.98448, -118.22681 | 3 |  |  | |  | |  |  |  | |  | |  |  |  |  |  |  |  |
|  |  |  |  |  |  | |  | |  |  |  | |  | |  |  |  |  |  |  |  |
|  | Malakwa, BC | 50.95443, -118.76123 | 3 |  |  | |  | |  |  |  | |  | |  |  |  |  |  |  |  |
|  |  |  |  |  |  | |  | |  |  |  | |  | |  |  |  |  |  |  |  |
|  | Mara, BC | 50.65385, -119.09082 | 3 |  |  | |  | |  |  |  | |  | |  |  |  |  |  |  |  |
|  |  |  |  |  |  | |  | |  |  |  | |  | |  |  |  |  |  |  |  |
| *Carabus nemoralis* (Müller) | Abbotsford, BC | 49.06429, -122.4037 | 3 |  |  | |  | |  |  |  | |  | |  |  |  |  |  |  |  |
|  |  |  |  |  |  | |  | |  |  |  | |  | |  |  |  |  |  |  |  |
|  | Revelstoke, BC | 50.98448, -118.22681 | 1 |  |  | |  | |  |  |  | |  | |  |  |  |  |  |  |  |
|  |  |  |  |  |  | |  | |  |  |  | |  | |  |  |  |  |  |  |  |
|  | Creston, BC | 49.09215, -116.46917 | 3 |  |  | |  | |  |  |  | |  | |  |  |  |  |  |  |  |
|  |  |  |  |  |  | |  | |  |  |  | |  | |  |  |  |  |  |  |  |
| *Pterostichus melanarius* (Illiger) | Abbotsford, BC | 49.06429, -122.4037 | 3 |  |  | |  | |  |  |  | |  | |  |  |  |  |  |  |  |
|  |  |  |  |  |  | |  | |  |  |  | |  | |  |  |  |  |  |  |  |
|  | Fort Steele, BC | 49.60934, -115.64057 | 1 |  |  | |  | |  |  |  | |  | |  |  |  |  |  |  |  |
|  |  |  |  |  |  | |  | |  |  |  | |  | |  |  |  |  |  |  |  |
|  | Mara, BC | 50.65385, -119.09082 | 1 |  |  | |  | |  |  |  | |  | |  |  |  |  |  |  |  |
|  |  |  |  |  |  | |  | |  |  |  | |  | |  |  |  |  |  |  |  |
|  | Revelstoke, BC | 50.98448, -118.22681 | 2 |  |  | |  | |  |  |  | |  | |  |  |  |  |  |  |  |
|  |  |  |  |  |  | |  | |  |  |  | |  | |  |  |  |  |  |  |  |
|  | Armstrong, BC | 50.41172, -119.19531 | 1 |  |  | |  | |  |  |  | |  | |  |  |  |  |  |  |  |
|  |  |  |  |  |  | |  | |  |  |  | |  | |  |  |  |  |  |  |  |
| *Calathus fuscipes* (Goeze) | Abbotsford, BC | 49.06429, -122.4037 | 3 |  |  | |  | |  |  |  | |  | |  |  |  |  |  |  |  |
|  |  |  |  |  |  | |  | |  |  |  | |  | |  |  |  |  |  |  |  |
| *Harpalus affinis* (Schrank) | Ladner, BC | 49.09640, -123.08456 | 1 |  |  | |  | |  |  |  | |  | |  |  |  |  |  |  |  |
|  |  |  |  |  |  | |  | |  |  |  | |  | |  |  |  |  |  |  |  |
|  | Vancouver, BC | 49.27580, -123.14537 | 2 |  |  | |  | |  |  |  | |  | |  |  |  |  |  |  |  |
|  |  |  |  |  |  | |  | |  |  |  | |  | |  |  |  |  |  |  |  |
|  | Revelstoke, BC | 50.98448, -118.22681 | 2 |  |  | |  | |  |  |  | |  | |  |  |  |  |  |  |  |
|  |  |  |  |  |  | |  | |  |  |  | |  | |  |  |  |  |  |  |  |
|  | Malakwa, BC | 50.95443, -118.76123 | 3 |  |  | |  | |  |  |  | |  | |  |  |  |  |  |  |  |
|  |  |  |  |  |  | |  | |  |  |  | |  | |  |  |  |  |  |  |  |
|  | Mara, BC | 50.65385, -119.09082 | 3 |  |  | |  | |  |  |  | |  | |  |  |  |  |  |  |  |
|  |  |  |  |  |  | |  | |  |  |  | |  | |  |  |  |  |  |  |  |
| *Agonum cupripenne* (Say) | Revelstoke, BC | 50.98448, -118.22681 | 4 |  |  | |  | |  |  |  | |  | |  |  |  |  |  |  |  |
|  |  |  |  |  |  | |  | |  |  |  | |  | |  |  |  |  |  |  |  |
|  | Malakwa, BC | 50.95443, -118.76123 | 2 |  |  | |  | |  |  |  | |  | |  |  |  |  |  |  |  |
|  |  |  |  |  |  | |  | |  |  |  | |  | |  |  |  |  |  |  |  |
|  | Revelstoke, BC | 50.98448, -118.22681 | 2 |  |  | |  | |  |  |  | |  | |  |  |  |  |  |  |  |
|  |  |  |  |  |  | |  | |  |  |  | |  | |  |  |  |  |  |  |  |
| *Amara littoralis* (Mannerheim) | Abbotsford, BC | 49.06429, -122.4037 | 3 |  |  | |  | |  |  |  | |  | |  |  |  |  |  |  |  |
|  |  |  |  |  |  | |  | |  |  |  | |  | |  |  |  |  |  |  |  |
|  | Windermere, BC | 50.46635, -115.97995 | 3 |  |  | |  | |  |  |  | |  | |  |  |  |  |  |  |  |
|  |  |  |  |  |  | |  | |  |  |  | |  | |  |  |  |  |  |  |  |
| **Scarabidae** |  |  |  |  |  | |  | |  |  |  | |  | |  |  |  |  |  |  |  |
| *Onthophagus nuchicornis* (Linnaeus) | Salmon Arm, BC | 50.67931, -119.21733 | 1 |  |  | |  | |  |  |  | |  | |  |  |  |  |  |  |  |
|  |  |  |  |  |  | |  | |  |  |  | |  | |  |  |  |  |  |  |  |
|  | Revelstoke, BC | 50.98448, -118.22681 | 3 |  |  | |  | |  |  |  | |  | |  |  |  |  |  |  |  |
|  |  |  |  |  |  | |  | |  |  |  | |  | |  |  |  |  |  |  |  |
|  | Creston, BC | 49.09215, -116.46917 | 3 |  |  | |  | |  |  |  | |  | |  |  |  |  |  |  |  |
|  |  |  |  |  |  | |  | |  |  |  | |  | |  |  |  |  |  |  |  |
| **Silphidae** |  |  |  |  |  | |  | |  |  |  | |  | |  |  |  |  |  |  |  |
| *Heterosilpha ramosa* (Say) | Salmon Arm, BC | 50.67931, -119.21733 | 1 |  |  | |  | |  |  |  | |  | |  |  |  |  |  |  |  |
|  |  |  |  |  |  | |  | |  |  |  | |  | |  |  |  |  |  |  |  |
|  | Revelstoke, BC | 50.98448, -118.22681 | 4 |  |  | |  | |  |  |  | |  | |  |  |  |  |  |  |  |
|  |  |  |  |  |  | |  | |  |  |  | |  | |  |  |  |  |  |  |  |
|  | Creston, BC | 49.09215, -116.46917 | 1 |  |  | |  | |  |  |  | |  | |  |  |  |  |  |  |  |
|  |  |  |  |  |  | |  | |  |  |  | |  | |  |  |  |  |  |  |  |
| *Nicrophorus investigator* (Zetterstedt) | Salmon Arm, BC | 50.67931, -119.21733 | 1 |  |  | |  | |  |  |  | |  | |  |  |  |  |  |  |  |
|  |  |  |  |  |  | |  | |  |  |  | |  | |  |  |  |  |  |  |  |
|  | Creston, BC | 49.09215, -116.46917 | 2 |  |  | |  | |  |  |  | |  | |  |  |  |  |  |  |  |
|  |  |  |  |  |  | |  | |  |  |  | |  | |  |  |  |  |  |  |  |
|  | Johnston Creek West, BC | 49.04614, -119.06191 | 3 |  |  | |  | |  |  |  | |  | |  |  |  |  |  |  |  |
|  |  |  |  |  |  | |  | |  |  |  | |  | |  |  |  |  |  |  |  |
|  | Revelstoke, BC | 50.98448, -118.22681 | 2 |  |  | |  | |  |  |  | |  | |  |  |  |  |  |  |  |
|  |  |  |  |  |  | |  | |  |  |  | |  | |  |  |  |  |  |  |  |
|  | Rock Creek, BC | 49.12939, -118.9936 | 1 |  |  | |  | |  |  |  | |  | |  |  |  |  |  |  |  |
