# Supplementary material for: PCR‐based species identification tools for wireworms (Coleoptera: Elateridae) of economic importance in Canada
Source: Pest Manag Sci. 2026 Jun 25;82(10):9998–10010. doi: 10.1002/ps.71036 (PMC13569292; doi:10.1002/ps.71036)
Supplement: Supplementary file 8 [file PS-82-9998-s008.docx]

**Supplementary Figure 4 –** Samples tested across a panel of target and non-target species from multiple geographic locations showing performance for each primer assay: (a) *Agriotes lineatus*, (b) *A. obscurus*, (c) *A. sputator* (d) *A. mancus*, (e) *A. pubescens,* (f) *A. ferrugineipennis*, (g) *Limonius agonus*, (h) *L. infuscatus*, (i) *L. californicus*, (j) *L. canus*, (k) *Selatosomus aeripennis destructor*, (l) *Hypnoidus bicolor*, (m) *H. abbreviatus*, (n) *Hadromorphus glaucus* and (o) *Athous haemorrhoidalis.* Box colour represents amplification type, with grey as a true negative (TN), green a true positive (TP), and red a false negative (FN). Zero false positives (FP) were found during testing. The text colour for a species and its corresponding collection site is black for Canadian populations and blue for populations from the United States of America (USA). Blocks of insect genus or family are annotated on the testing panel. False negatives were only found for (i) *L. californicus* and (h) *L. infuscatus* primer assays for populations tested in the USA.
